# A Developmental Profile of Children With Autism Spectrum Disorder in China Using the Griffiths Mental Development Scales

**DOI:** 10.3389/fpsyg.2020.570923

**Published:** 2020-11-09

**Authors:** Hong-Hua Li, Cheng-Xin Wang, Jun-Yan Feng, Bing Wang, Chun-Li Li, Fei-Yong Jia

**Affiliations:** ^1^Department of Developmental and Behavioral Pediatrics, The First Hospital of Jilin University, Changchun, China; ^2^Pediatric Research Institute, Changchun, China

**Keywords:** autism spectrum disorder, children, developmental assessment, griffiths mental development scales, mental development

## Abstract

The purpose of this study was to profile the mental development of children aged 18 to 96 months with autism spectrum disorder (ASD) using the Chinese version of the Griffiths Mental Development Scales (GMDS), and to explore the relationships between developmental levels and ASD severity, the sex of the child and the age of ASD diagnosis. Children with ASD (*n* = 398; 337 boys, 61 girls) were recruited and ASD severity evaluated using the Autism Behavior Checklist and the Childhood Autism Rating Scale, while the GMDS was used to evaluate the children’s mental development. Study participants were divided into groups according to GMDS general and subscale quotients, ASD severity, sex, and age. The majority of groups divided according to the GMDS quotients exhibited an unbalanced distribution in respect of the six domains of the GMDS and there were significant differences within the six subscale quotients. Autism severity, sex and age had significant effects on the overall level of development of autistic children. The quotients recorded for the children with more severe ASD were significantly lower than those for the children with less severe ASD. A markedly higher proportion of developmental delay was recorded for girls than boys in relation to the performance subscale. The locomotor quotient decreased in line with age at diagnosis, while autism severity and age had significant effects on the general and subscale quotients and sex had a significant effect on performance quotient. Children with ASD exhibit an uneven cognitive development profile, and their overall developmental levels are affected by autism severity, sex and age. Specific cognitive domains differ according to sex in children with ASD. Locomotor skills tend to decrease according to the age at diagnosis for autistic children aged 18 to 84 months. Autism severity and age are also associated with the level of functioning in different cognitive areas. These findings contribute to define the cognitive developmental profiles of children with ASD.

## Introduction

Autism spectrum disorder (ASD) is a neurodevelopmental condition, and individuals with the condition typically exhibit a range of atypical social interactions, communication difficulties, the presence of repetitive and stereotyped behavior, and restricted interests. The worldwide prevalence of the condition is thought to be between 1% and 3% of the general population, with a proportional distribution of four or five males to one female (American Psychiatric Association, 2013; Christensen et al., 2019). The sex imbalance in prevalence may be related to the underlying neurobiological mechanism. In addition, there may be sex differences in the autistic symptoms and cognitive development level of children with ASD, leading to the under-recognition and under-diagnosis of girls with ASD, exaggerating the sex imbalance (Van Wijngaarden-Cremers et al., 2014; Carpenter et al., 2019). Some community-based studies indicated that the true estimate of the ratio is likely to be closer to 3:1 (Honda et al., 2005; Kim et al., 2011; Idring et al., 2012).

The etiological factors remain largely unknown, but epigenetic factors, such as histone modification, DNA methylation and non-coding RNA, and the gut–microbiota–brain axis have been theorized to play an important role in ASD etiology (Martinez-Gonzalez and Andreo-Martinez, 2019; Andreo-Martinez et al., 2020; Yoon et al., 2020). Individuals with ASD often experience additional developmental disorders, with roughly 30% of those with ASD exhibiting other intellectual disabilities (IDs) (American Psychiatric Association, 2013). Children with more severe ASD (the nature and extent of ASD-related characteristics, henceforth referred to as the severity of ASD symptoms) generally have lower social adaptation abilities and require more support (Gardner et al., 2018). Additionally, autistic children with low developmental levels require more early intensive intervention to promote their developmental progress (Hinnebusch et al., 2017), and children with ASD who do not receive diagnoses of IDs and other developmental disorders may experience poorer developmental outcomes (Miller et al., 2019).

Children with ASD often exhibit unbalances with respect to their cognitive processing according to developmental assessment (Li et al., 2019). For example, compared with typically developing children, those with ASD can show significant difficulties concerning their relational and phonological working memory capacities (Ring et al., 2016; Habib et al., 2019). Their local visual information processing may be superior to that of typically developing children, but no differences between the two groups have been found with regard to global visuospatial performance (Muth et al., 2014; Nilsson Jobs et al., 2018). In addition, there may be sex differences in terms of the relative cognitive structures or developmental levels of individuals with ASD. Abilities corresponding to visual attention to detail in boys described as having high-functioning ASD were found to be superior to those of girls in Bölte et al.’s (2011) study, while Matheis et al. (2019) observed that girls aged 17–37 months who had ASD exhibited less communication difficulty but greater motor challenges compared to boys in the same age group. A further consideration is that the relative developmental levels of children with ASD may be related to the age at which they are diagnosed. For instance, Licari et al. (2020) analyzed the motor abilities of 2,084 autistic children younger than 6 years old, and found that approximately 30% of the children met the criteria for motor difficulties, and that the prevalence of motor difficulties increased with the age of diagnosis.

The Autism Diagnostic Interview–Revised (Rutter et al., 2003), the Autism Diagnostic Observation Schedule (ADOS) (Lord et al., 2000), and the Diagnostic Interview for Social and Communication Disorders (Wing et al., 2002) are the diagnostic tools that are most commonly used in relation to ASD (Randall et al., 2018). Children diagnosed with ASD may need to choose different types of educational centers, according to the severity of ASD symptoms and their cognitive levels. Therefore, before receiving intervention or education, they will require a standardized overall developmental assessment, with the purpose of refining the clinical diagnosis. More importantly, children’s relative strengths and weaknesses can be identified at this stage, in order to facilitate the development of a constructive personalized intervention plan.

The Chinese versions of the Wechsler Primary and Preschool Scale of Intelligence (Wechsler, 2002) and Wechsler Intelligence Scale for Children (Wechsler, 2004) are commonly used intelligence tests in China, but they are only applicable to children aged between four and a half and 16 years old, and are not suitable for use in evaluating younger children (Gong and Dai, 1988; Yang, 2016). The Gesell Developmental Schedule, the Children’s Neuropsychological and Behavior Scale, and the Griffiths Mental Development Scales (GMDS) are all diagnostic assessment tools commonly used in China to evaluate the development of children aged up to 6–8 years old. Of these, though, while the original version of the Gesell Developmental Schedule has been refined and updated, it has not been revised in the past 20 years for use in Chinese contexts (Yang, 2016), and the Children’s Neuropsychological and Behavior Scale is a local assessment tool in China for which only the Chinese psychometric properties are currently available (Li et al., 2019).

Accordingly, the GMDS is the instrument that is generally used for evaluating developmental progress in children from birth up to 8 years of age (Luiz et al., 2001). It can be utilized for many clinical applications, such as predicting future developmental outcomes or evaluating the impact of epilepsy, antiepileptic drugs, and congenital heart disease surgery on infants’ cognitive development (Dittrich et al., 2003; Randò et al., 2005; Bromley et al., 2010; Doyle et al., 2012). Moreover, the GMDS can be utilized as an intelligence test, in that the overall score (“general quotient”) corresponds to an IQ score, and so it can be used to investigate the prevalence of ID in autistic children (Postorino et al., 2016; Scandurra et al., 2019).

Age at diagnosis, degree of atypicality, and level of intelligence may be key factors in predicting long-term developmental outcomes for individuals with ASD (Coplan and Jawad, 2005). Consequently, it is important to assess developmental levels in preschool and in early childhood. Prior studies have been primarily concerned with the cognitive characteristics of school-age children and adults with ASD, and the Wechsler (2002, 2004) intelligence scales have been most often used in such research (Hidding et al., 2015; Kanai et al., 2017; Kim and Song, 2020). Although some previous studies have used the GMDS as an intelligence tool with which to assess the prevalence of ID in autistic children (Postorino et al., 2016; Scandurra et al., 2019), few have reported the motor, language, social, and reasoning abilities of children with ASD through reference to the GMDS subscale, or how these levels of ability correlate with the autism severity, age, and sex of the assessed individual.

The Chinese version of the GMDS was revised for use with Chinese children in 2016, based on the 2006 update to the 1996 version of the GMDS (Luiz et al., 2006). A cross-cultural comparison study confirmed that GMDS was well adapted to a Chinese context and could reliably be used to assess development in Chinese children from birth to 8 years old (Tso et al., 2018). Li et al. (2020) found that the scale had good reliability and validity in the evaluation of children aged 3 to 8 years old with ASD. Recently, He et al. (2019) used the scale to assess the mental development of children with ASD in China–specifically, to analyze the correlation between the developmental levels and eye movement characteristics of 21 preschoolers with ASD. To date, there is currently a dearth of literature characterizing the cognitive motor and social profiles of autistic children in China. The purpose of the present study is to profile the developmental levels of 398 children (18 to 96 months old) with ASD across the different areas of the GMDS, in order to explore whether there are unbalances between these areas, and to analyze the correlations between the developmental levels measured and the severity of the ASD, the sex of the assessed child, and the age at which they were first diagnosed with ASD as an attempt to provide a theoretical basis for interventions and educational decision-making in respect of children with ASD.

## Materials and Methods

### Participants

The study’s participants were recruited from a group of children who had exhibited signs of ASD and were being evaluated for the first time at the Child Developmental and Behavioral Division of the First Hospital of Jilin University in Changchun, China, during the period March 2018 to December 2019. Initially, all of the children with suspected ASD were examined through reviews of their current health, developmental history, and family history, as well as through a clinical physical examination and parental interviews carried out by at least two developmental pediatricians with reference to the *Diagnostic and Statistical Manual of Mental Disorders* (DSM-5) criteria (American Psychiatric Association, 2013). In addition, an ADOS administration was undertaken by trained developmental pediatricians. Ultimately, 398 children from this group were found to fulfill the DSM-5 criteria for ASD and had positive results from their ADOS administration.

All participants in the study completed assessments designed to evaluate both their developmental levels and the severity of their ASD symptoms. The children had a mean age of 41.6 ± 15.6 months (range: 18–96 months), and the overall study cohort was composed of 337 boys and 61 girls with a 5.5:1 male-to-female ratio. All children were examined for common comorbidities such as epilepsy, and, following comprehensive medical observation and neuroimaging, genetic metabolism, chromosome, and other related examinations, we excluded children with Rett syndrome, fragile X syndrome, genetic metabolic disorders, and other neurological conditions such as epilepsy.

In the past, we have published outcomes from analyses of 114 boys and 25 girls enrolled in this study (Li et al., 2019). Prior to participation, all of the legal guardians of the children with ASD had given written informed consent. The Ethics Committee of the First Hospital of Jilin university approved this study (No: 2017-314).

### Measurements

#### Evaluation of ASD Symptoms

The severity of ASD symptoms in the children participating in this study was assessed by a developmental pediatrician using the Autism Behavior Checklist (ABC) (Krug, 1980) and the Childhood Autism Rating Scale (CARS) (Schopler et al., 1980). The ABC and the CARS are both commonly used assessment scales in China in clinical practice and ASD research.

The ABC is an unstructured behavior questionnaire that is completed by the child’s parent or caregiver. The checklist features 57 items, covering five aspects of atypical behavior: sensory, relating, body concept and object use, language, and self-care. The score for each item ranges from 1 to 4, and total scores for the ABC range from 0 to 158, with higher scores indicating increased levels of ASD symptoms. A typically developing child’s ABC score should be less than 47 (Krug, 1980; Krug et al., 1993). The Chinese version of the ABC has been found to have good psychometric properties (Yang et al., 1993), and with a cut-off score of 50 of the checklist, autism was screened from the normal population with a sensitivity of 0.97 and a specificity of 1 (Yang et al., 1993).

The CARS is a 15-item observational scale. Each item was graded by the developmental pediatrician on the basis of the symptom criteria, with a rating of 1 denoting “*normal*,” 2 “*mild*,” 3 “*moderate*,” and 4 “*severe*.” Typically developing children exhibit CARS scores of lower than 30 (Schopler et al., 1980), and higher scores indicate more severe ASD symptoms. The reliability coefficient (Cronbach’s alpha) was 0.94, and the correlation coefficient between the scale scores and clinicians’ ratings was 0.80, indicating good reliability and validity for the CARS (Schopler et al., 1980).

#### ASD Diagnostic Evaluation

In order to further corroborate the diagnosis of ASD, all children with suspected ASD underwent an ADOS assessment, performed by a developmental pediatrician who had received training and qualified in ADOS evaluation. In this study, the Chinese version of the ADOS was used, which was revised based on the second edition of ADOS (ADOS-2; Lord et al., 2012). The ADOS is a play-based, semi-structured assessment tool for assessing current autistic behaviors. It consists of four different modules, which are selected by the child undergoing assessment according to their current expressive language level. Each module has a specific diagnostic algorithm for two domains: social affect and restricted and repetitive behavior. Overall, an ADOS evaluation takes about 45 min to complete. The total score of each module has a cut-off point corresponding to whether it conforms to the diagnosis of ASD.

#### Assessment of Mental Development

The GMDS was used to evaluate the developmental levels of the autistic children. Three experienced developmental pediatricians who had been formally trained in the test and were qualified to use it for research evaluation participated in this study. The Chinese version of the GMDS was revised based on the 2006 version of the GMDS and featured normative data relating to China (Luiz et al., 2006; Tso et al., 2018).

The GMDS measures a child’s abilities through reference to the following six subscales: subscale A is the “locomotor” scale, measuring movement with regard to graded coordination, economy of effort, and postural control; subscale B measures “personal–social” abilities, covering growing self-awareness, independence, and social interaction; subscale C assesses “hearing and language,” rating the child’s ability to hear, listen, and comprehend, as well as to express themselves; subscale D appraises “eye and hand coordination,” or visual competence with fine motor precision functionality; subscale E covers “performance” as it pertains to visual perception awareness, including working speed and precision; and subscale F corresponds to “practical reasoning,” or a 2 to 8-year-old child’s ability to use past learning experiences to solve problems, as well as their understanding of basic mathematical concepts and moral issues.

The mean of the general quotient (GQ) and each of the six subscale quotients is 100 points (*SD* = 15 points). The subscale quotients are calculated using the developmental age corresponding to each subscale divided by the actual chronological age and multiplying by 100. The GQ raw score is the sum of the subscales raw scores. A GQ or a subscale quotient <70 points (>2SD below the mean) is considered to indicate a significant delay in development, while a quotient >70 points indicates a mild or no delay (Cirelli et al., 2015). The Cronbach’s alpha of the full scale of the Chinese version of the GMDS was 0.98, indicating a strong correlation between the subscales, while the subscales’ Cronbach’s alphas were all above 0.7, suggesting acceptable internal consistency (Tso et al., 2018).

### Procedures

During the first visit to the hospital, children who had exhibited signs of ASD will receive an initial assessment of approximately 20 min by an outpatient developmental behavioral pediatrician, including current health, developmental history, and family history. For children suspected with ASD, the outpatient pediatrician would schedule an evaluation checklist containing ABC, CRAS, GMDS, and ADOS. On the day of the first visit, the parents of the participating children completed the ABC after being given instructions on how to do so by a developmental pediatrician in the evaluation room of the Child Developmental and Behavioral Division. At the same time, the pediatrician completed the CARS by observing the child’s behavior and conducting an interview with their parent or guardian. If the child was in a good condition, a trained and qualified developmental pediatrician would complete the GMDS on the day of the first visit too. The GMDS assessment was performed in a quiet examination room or a training room approximately 20 square meters in size and with no distracting objects in the room during the course of the evaluation. A full GMDS evaluation takes approximately one and a half hours to complete. If a child had an obvious emotional reaction during the evaluation, a new appointment could be made, but the evaluation had to be completed within 1 week. The ADOS was also usually completed within 1 week, with the certified developmental pediatrician completing it in an assessment room of approximately 20 square meters in size, and a full ADOS assessment took approximately 45 min for each child.

### Statistical Analyses

The data were analyzed using SPSS Statistics, version 22.0 (IBM Corp., NY, United States). The normality of the data was analyzed using the Kolmogorov–Smirnov test. Continuous data were means ± *SD*s or P50(P25, P75) (i.e., median, 25th percentile, and 75th percentile measures), whereas categorical data were given as frequencies with percentages. Based on the GQ and five subscale quotients (i.e., those for the GMDS subscales B to F; subscale A was excluded for the weak correlation with cognitive structure), the study’s sample of autistic children was subdivided into two groups, as follows. Children who received a GQ or a subscale quotient >70 points were assigned to a *higher developmental-level* group, while children who scored <70 points were allocated to a *lower developmental-level* group. Subjects in the former group were observed to have demonstrated mild or no delay in their general development or in one of the domains measured by the GMDS, whereas children in the latter group had exhibited a significant delay in terms of their general development or in respect of one of the domains measured by the GMDS. The Kruskal–Wallis *H* test was used to compare the differences of quotients in various fields of the GMDS within each group. Mixed ANOVA was used to compare the mean differences within the six subscales (GQ and subscale A to E), as well as differences in overall level of development among factors such as autism severity, sex and age.

According to their total CARS scores, children with ASD were assigned into groups as follows. Children with a total CARS score of fewer than 32 points were considered to have *less severe* ASD (*n* = 226, mean CARS score = 28), whereas ASD was considered to be *more severe* in children whose scores were equal to or higher than 32 points (*n* = 172, mean CARS score = 35). A 32-point cutoff was chosen as it was the mean CARS score for all of children with ASD in the study’s sample. An independent samples *t*-test and a non-parametric Mann–Whitney *U* test were used to compare the continuous data of the two groups. Normally and non-normally distributed data were analyzed using Pearson’s and Spearman’s correlation coefficient tests, respectively.

An independent samples *t*-test, a non-parametric Mann–Whitney *U* test, and chi-squared tests were used for comparing variables between the different sex subgroups. Cohen’s *d* was calculated between the variables to represent the magnitude of the differences. The Kruskal–Wallis *H* test was also used to compare the differences of the GQ and subscale quotients of the GMDS among the six age groups.

Multi-way ANOVA was used to compare the effects of autism severity, sex and age on the GQ and six subscale quotients of the GMDS. All tests were two-sided, with *P* < 0.05 as the significance threshold.

## Results

### Autism Severity and Developmental Levels of the Participants

The total ABC score of the participants was 51.7 ± 16.9, and the total CARS score was 31.5 ± 4.4. The subscales and GQ scores of the GMDS of the participants are summarized in Table 1. Developmental delays were considered to be present when a GQ or subscale quotient was at least 2 *SD* below the mean (a GQ or a subscale quotient <70). Data for children exhibiting developmental delays in the different domains of this scale are presented in Table 1. As these children had limited language ability and developmental progression, measurable practical reasoning scores (i.e., obtained using the GMDS subscale F) were only available for 99 children (25%); no scores in this domain were provided for the other 299 children (75%).

**TABLE 1:** GMDS assessment results for children diagnosed with ASD by the age of 8 (96 months)^*a*^.

Quotient (subscale letter label)	Mean ± *SD*	Delay^b^ *n* (%)
General (GQ)	62.2 ± 17.2	279 (70.1%)
Locomotor (AQ)	75.7 ± 17.9	146 (36.7%)
Personal–social (BQ)	57.4 ± 19.3	297 (74.6%)
Hearing and language (CQ)	48.0 ± 23.0	332 (83.4%)
Eye–hand coordination (DQ)	63.3 ± 19.0	251 (63.1%)
Performance (EQ)	66.7 ± 23.4	227 (57.0%)
Practical reasoning (FQ)^c^	70.7 ± 23.4	56/99 (56.6%)
Delayed in two or more domains	–	316 (79.4%)

*^a^N = 398. ^b^A GQ or a subscale quotient <70. ^c^FQ was measured for 99 children (2–8 year olds) in the sample.*

### The Developmental Profiles of the Participants in the Two Developmental Level Categories

**FIGURE 1:**
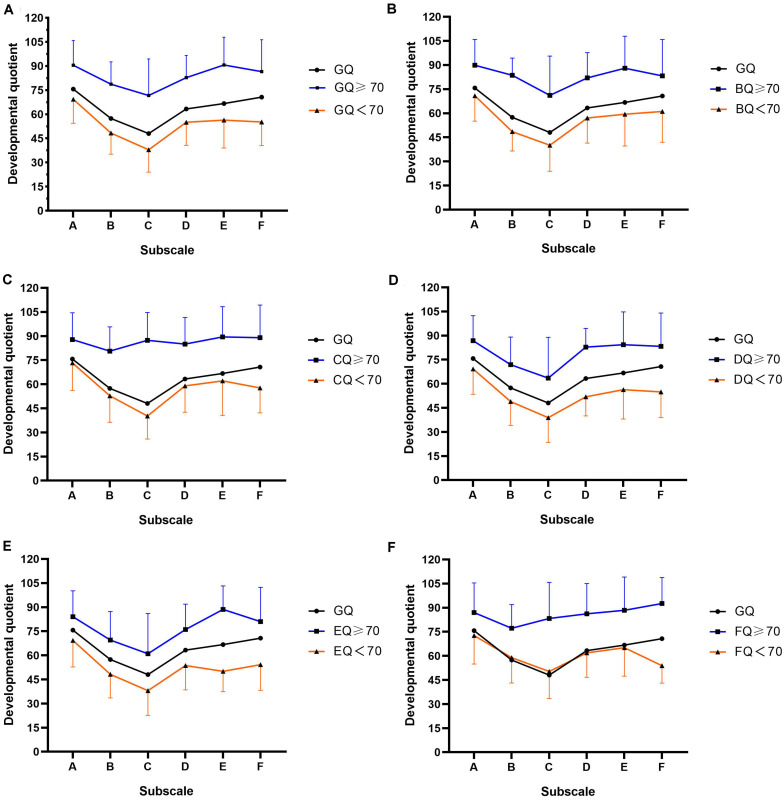
Developmental profiles of autistic children included in this study plotted according to the two categorized developmental levels and grouped by GQ or subscale quotients. **(A)** Mean GMDS subscale GQ scores for the whole group and for each developmental level. Mean GQ score and mean developmental quotients on the subscales in the two levels grouped by: **(B)** GMDS subscale B (personal–social, BQ), **(C)** GMDS subscale C (hearing and language, CQ), **(D)** GMDS subscale D (eye–hand coordination, DQ), **(E)** GMDS subscale E (performance, EQ), **(F)** GMDS subscale F (practical reasoning, FQ). Standard deviations of the mean are represented in the figure by the error bars attached to each line of the two developmental levels.

Figure 1 plots the developmental profiles of the study’s sample of autistic children in the two developmental level categories, grouped by GQ or subscale quotients. As can be seen in Figures 1A,B,D,E, participants in both the higher and the lower developmental-level groups (grouped by GQ, BQ, DQ, and EQ, respectively) demonstrated an unbalanced distribution of GMDS results in six areas. The relatively best results were found in relation to the locomotor and performance subscales (i.e., A and E, respectively) while the lowest was in respect of the hearing and language subscale (C). However, as illustrated in Figures 1C,F, the developmental quotient distribution curves of the higher developmental-level groups (grouped by CQ and FQ, respectively) were relatively flat. There were no significant differences observed among the six subscale quotients in the higher developmental-level group of Figure 1C (*n* = 66, *H* = 4.862, *P* = 0.433), indicating that there was no developmental imbalance found for the children in this group. In the higher developmental-level group of Figure 1F, except for BQ (mean = 77.2, *SD* = 14.7), no significant differences were found among the remaining subscale quotients (*n* = 43, *H* = 2.420, *P* = 0.659).

### Differences Within Subscales of GMDS and the Effects of Autism Severity, Sex and Age on Overall Developmental Level

A 2(autism severity) × 2(sex) × 7 (chronological age group) × 6(subscales) ANOVA gave a significant difference within the six subscale quotients of the GMDS (*F* = 43.191, *P* < 0.001, η*^2^* = 0.359), indicating an unbalanced distribution of GMDS results in six domains of the autistic children. The test also gave significant major effects of autism severity (*F* = 6.819, *P* < 0.001, η*^2^* = 0.081), sex (*F* = 3.188, *P* = 0.008, η*^2^* = 0.040) and age (*F* = 12.252, *P* < 0.001, η*^2^* = 0.159) on the overall level of development of autistic children. Age and autism severity (*F* = 2.138, *P* = 0.048, η*^2^* = 0.033) had interacting effects on the overall level of development; however, sex and age (*F* = 2.040, *P* = 0.072, η*^2^* = 0.027), sex and autism severity (*F* = 0.175, *P* = 0.972, η*^2^* = 0.002) had no interacting effects.

### Developmental-Level Differences With Less Severe Versus More Severe ASD

The developmental levels of children with ASD of different levels of severity are detailed in Table 2. The ages at diagnosis of those in the more severe group were significantly lower than those in the less severe group. No significant differences in sex between the two groups. Total ABC scores and total CARS scores of children in the more severe group were significantly higher than those of the less severe group. The GQ and six mean or median subscale quotients (AQ–FQ) recorded for children with more severe ASD levels were significantly lower than those for children with less severe ASD levels.

**TABLE 2:** Developmental levels in children with less severe versus more severe ASD.

Variable	Less severe^a^	More severe^b^	*t* (*Z*)/χ^2^	*P*
Age at diagnosis (months)	43.8 ± 15.6	38.7 ± 15.3*	3.28	0.001
Sex Boys Girls	198(87.6%) 28(12.4%)	139(80.8%) 33(19.2%)	3.477	0.062
Total ABC score	43.5 ± 14.6	62.5 ± 13.1*	13.4	<0.001
Total CARS score	28.4 ± 2.9	35.4 ± 2.2*	26.13	<0.001
General (GQ)	68.6 ± 16.5	53.7 ± 14.4*	9.38	<0.001
Locomotor (AQ)	79 ± 17.9	71.3 ± 16.9*	4.38	<0.001
Personal–social (BQ)	65.1 ± 17.6	47.2 ± 16.5*	10.33	<0.001
Hearing and language (CQ)	56.4 ± 23.5	36.9 ± 16.8*	9.23	<0.001
Eye–hand coordination (DQ)	69.6 ± 18.1	55.1 ± 17.0*	8.15	<0.001
Performance (EQ)	73.3 ± 22.3	57.9 ± 22.0*	6.86	<0.001
Practical reasoning (FQ)^c^	68 (55, 92)	57 (37, 76)*	(2.17)	0.03

^a^n = 226. ^b^ n = 172. ^c^FQ was measured for 99 children (2–8 year olds) in the sample: less severe, 83; more severe, 16. * Significantly different from values obtained for the less severe ASD group, P < 0.05.

Table 3 presents the correlation coefficients of the total ABC and CARS scores alongside age at diagnosis and the GMDS GQ and subscale quotients. Total ABC and CARS scores were found to be negatively correlated with age at diagnosis, GQ, and the subscale quotients.

**TABLE 3:** Correlation of total ABC and CARS scores with age at diagnosis and GMDS general and subscale quotients.

Variable	Total ABC score *r*_*s*_/(*r*)	Total CARS score *r*_*s*_
Age at diagnosis (months)	−0.19**	−0.21**
General (GQ)	(−0.38)**	−0.54**
Locomotor (AQ)	(−0.21)**	−0.26**
Personal−social (BQ)	(−0.45)**	−0.58**
Hearing and language (CQ)	(−0.34)**	−0.56**
Eye−hand coordination (DQ)	(−0.34)**	−0.48**
Performance (EQ)	(−0.27)**	−0.40**
Practical reasoning (FQ)	−0.21*	−0.41**

Spearman’s rank correlation coefficient was used to obtain r_s_; the Pearson correlation coefficient was used to obtain r. *The correlation was significant, P < 0.05. **The correlation was significant, P < 0.01.

### Sex Differences for Autism Severity and Developmental Levels

Relative levels of autism severity and the developmental quotients of children with ASD for both sexes are compared in Table 4. No significant differences were found in relation to age at diagnosis or total ABC and CARS scores between boys and girls in the study’s sample. However, the GQ of boys was significantly higher than that of girls. Regarding the six subscale quotients, the DQ, EQ, and FQ of boys were found to be significantly higher than those of girls, but no significant differences in AQ, BQ, or CQ were recorded between the two sexes.

**TABLE 4:** Sex differences in autism severity and developmental levels of children with ASD^*a*^.

Item	Boys^*b*^	Girls^*c*^	*t* (*Z*)	Cohen’s *d*	*P*
Age at diagnosis (months)	41.5 ± 14.7	42.4 ± 19.9	0.326	0.05	0.746
Total ABC score	51.6 ± 16.7	52.6 ± 18.1	0.422	0.06	0.673
Total CARS score	31.3 ± 4.3	32.1 ± 4.7	1.011	0.18	0.312
General (GQ)	63.1 ± 16.7*	57.2 ± 19.5	2.440	0.33	0.015
Locomotor (AQ)	76 ± 17.3	73.8 ± 20.8	0.893	0.12	0.372
Personal–social (BQ)	58.1 ± 18.5	53.6 ± 22.8	1.649	0.22	0.100
Hearing and language (CQ)	48.4 ± 23.1	45.7 ± 22.7	0.855	0.12	0.393
Eye–hand coordination (DQ)	64.4 ± 18.3*	57.3 ± 21.9	2.704	0.35	0.007
Performance (EQ)	68.5 ± 23*	56.7 ± 23.4	3.656	0.51	<0.001
Practical reasoning (FQ)^*d*^	68 (55, 89)*	52 (37, 63)	(2.228)	–	0.026

^a^P50(P25, P75), mean ± SD. ^b^n = 337. ^c^n = 61. ^d^FQ was measured for 99 children (2–8 year olds) in the sample: 91 boys and 8 girls. *Significantly different from values obtained for the girls, P < 0.05.

The proportions of children of both sexes exhibiting delays in the different domains of the GMDS are summarized in Table 5. The proportions of children with developmental delays recorded in relation to the general, locomotor, personal–social, hearing and language, eye–hand coordination, and practical reasoning subscales did not differ significantly between boys and girls. However, the proportion of boys who were observed to have a developmental delay with reference to the performance subscale (EQ < 70) was significantly lower than that of girls.

**TABLE 5:** Sex differences in the numbers of children exhibiting developmental delays based on GMDS quotients.

Subscale of GMDS (quotient letter label)	Boys^a^ *n* (%)	Girls^b^ *n* (%)	χ^2^ *n* (%)	*P* *n* (%)
General (GQ)	234 (69%)	45 (73%)	0.463	0.496
Locomotor (AQ)	125 (37%)	21 (34%)	0.158	0.691
Personal–social (BQ)	251 (74%)	46 (75%)	0.024	0.878
Hearing and language (CQ)	278 (82%)	54 (89%)	1.359	0.244
Eye–hand coordination (DQ)	208 (62%)	43 (70%)	1.706	0.192
Performance (EQ)	181 (53.7%)*	46 (75%)	9.926	0.002
Practical reasoning (FQ)^c^	49/91 (54%)	7/8 (88%)	3.390	0.067

^a^n = 337. ^b^n = 61. ^c^FQ was measured for 99 children (2–8 year olds) in the sample: 91 boys and 8 girls. *Significantly different from values obtained for the girls, P < 0.05.

### Distribution of the Subscale Quotients of GMDS in Different Age Groups

We also analyzed variations in the developmental quotients of the GMDS in relation to the children’s different age groups. There were only eight children (3 boys, 5 girls) in the sample aged between 84 and 96 months (i.e., 7 and 8 years old), and only 99 children were measured in relation to the practical reasoning domain (subscale F, which only applied to children between 2 and 8 years old). Accordingly, in order to avoid data bias caused by small sample sizes, these two groups were excluded from the data.

**FIGURE 2:**
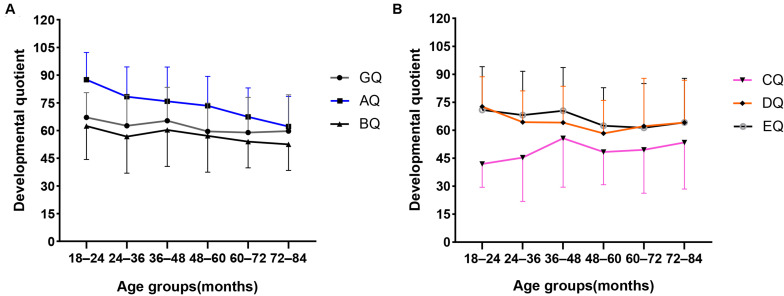
Mean scores distribution of the GQ and A–E subscale quotients in different age groups. **(A)** Mean scores distribution of the GQ, AQ, and BQ in different age groups. **(B)** Mean scores distribution of the CQ, DQ, and EQ in different age groups. Standard deviations of the mean are represented in the figure by the error bars attached to each line.

Table 6 shows the GQ and subscale quotients of the GMDS across six age groups. There were no statistically significant differences found regarding the mean scores of the GQ, BQ, and EQ between the six age groups. However, the mean scores of the AQ, CQ, and DQ were statistically significant between the different age groups. In addition, as can be seen in Figure 2, the mean scores of the AQ decreased with age at diagnosis, and there was a significant negative correlation between AQ and age at diagnosis (Figure 2) (*r* = −0.310, *P* < 0.001); however, there was no decreasing trend corresponding to age at diagnosis in the mean CQ or the DQ mean scores. With reference to the hearing and language subscale, the highest mean scores of CQ were for the 36–48 months group, and the lowest were for the 18–24 months group. In respect of the eye–hand coordination subscale, the highest scores for the DQ were in the 18–24 months group, while the mean scores of the remaining age groups fluctuated within the range of 58.3 to 64.3 points.

**TABLE 6:** GMDS general and subscale quotients of in different age groups (months)^a^.

Quotient	18–24^b^	24–36^c^	36–48^d^	48–60^e^	60–72^f^	72–84^g^	*H*	*P*
GQ	67.1 ± 13.4	62.6 ± 16.5	65.3 ± 18.1	59.5 ± 15.3	58.9 ± 19.0	59.7 ± 19.6	9.483	0.091
AQ*	87.5 ± 14.8	78.3 ± 16.2	75.8 ± 18.6	73.4 ± 15.9	67.4 ± 15.7	62.2 ± 16.3	41.227	<0.001
BQ	62.4 ± 18.1	56.7 ± 19.8	60.3 ± 19.7	57.1 ± 19.6	54.0 ± 14.2	52.5 ± 14.1	6.037	0.303
CQ*	41.9 ± 12.5	45.3 ± 23.5	55.7 ± 26.2	48.3 ± 17.5	49.5 ± 23.3	53.4 ± 24.9	17.632	0.003
DQ*	72.7 ± 16.0	64.3 ± 16.8	64.1 ± 19.5	58.3 ± 17.7	62.2 ± 25.6	64.1 ± 22.7	15.133	0.01
EQ	70.9 ± 23.2	68.1 ± 23.5	70.4 ± 23.2	62.4 ± 20.4	61.3 ± 23.7	64.2 ± 23.6	7.186	0.207

^a^Mean ± SD. ^b^n = 34. ^c^n = 163. ^d^n = 92. ^e^n = 60. ^f^n = 27. ^g^n = 14. *Significantly different among the six age groups, P < 0.05.

### The Effects of Autism Severity, Sex and Age on the GMDS General and Subscale Quotients

The effects of autism severity, sex and age on the GQ and six subscale quotients of the GMDS using multi-way ANOVA test were detailed in Table 7. Autism severity had significant major effects on GQ and subscale quotients except FQ. Age had significant major effects on the general and six subscale quotients of GMDS. Sex had a significant major effect on EQ. No interacting effects were found among factors of autism severity, sex and age.

**TABLE 7:** Effects of autism severity, sex and age on the GMDS general and subscale quotients.

Variable	GQ	AQ	BQ	CQ	DQ	EQ	FQ^*a*^
Autism severity	*F* = 103.854 *P*<0.001	*F* = 36.379 *P*<0.001	*F* = 118.856 *P*<0.001	*F* = 75.983 *P*<0.001	*F* = 83.906 *P*<0.001	*F* = 53.642 *P*<0.001	*F* = 2.94 *P* = 0.09
Sex	F = 1.238 *P* = 0.267	*F* = 0.011 *P* = 0.915	F = 0.390 *P* = 0.533	*F* = 0.574 *P* = 0.449	*F* = 2.952 *P* = 0.087	*F* = 6.984 *P* = 0.009	*F* = 1.300 *P* = 0.257
Age (group)	*F* = 7.832 *P*<0.001	*F* = 15.121 *P*<0.001	*F* = 4.546 *P*<0.001	*F* = 3.094 *P* = 0.006	*F* = 8.415 *P*<0.001	*F* = 5.015 *P*<0.001	*F* = 4.053 *P* = 0.002
Sex* Autism severity	*F* = 0.476 *P* = 0.505	*F* = 1.384 *P* = 0.264	*F* = 0.078 *P* = 0.788	*F* = 0.450 *P* = 0.509	*F* = 0.309 *P* = 0.588	*F* = 0.313 *P* = 0.582	–
Sex*Age	*F* = 0.605 *P* = 0.703	*F* = 1.711 *P* = 0.285	*F* = 0.141 *P* = 0.975	*F* = 1.214 *P* = 0.418	*F* = 1.502 *P* = 0.333	*F* = 0.162 *P* = 0.966	–
Age*Autism severity	*F* = 1.911 *P* = 0.233	*F* = 0.746 *P* = 0.636	*F* = 1.475 *P* = 0.336	*F* = 3.603 *P* = 0.064	*F* = 2.461 *P* = 0.153	*F* = 1.882 *P* = 0.226	–

*Independent variables: Autism severity (less severe and more severe group); Sex (boys and girls); Age (seven age groups). ^a^In some age groups, there was only one girl with FQ, so the interacting effects on FQ cannot be calculated.*

## Discussion

This study set out to profile the mental development of children with ASD between the ages of 18 and 96 months old. The relationships between developmental level and autism severity, sex, and age at diagnosis were also explored. Nearly 80% of the children included in this study were found to have comorbid developmental delays concerning two or more domains of the GMDS, a finding that is consistent with prior studies that have discovered that the majority of individuals with ASD have mild to moderate IDs, along with language difficulty (Postorino et al., 2016; Narzisi et al., 2018). In addition, similar to other studies, we found that most of the children exhibited a cognitive profile that typically encompassed uneven cognitive development, with relative strengths with regard to the locomotor and performance domains and weaknesses in respect of the hearing and language domain (Sandberg et al., 1993; Joseph et al., 2002); however, in the higher developmental-level group in this study, results pertaining to the hearing and language subscale (CQ > 70) and the six domains of the GMDS were relatively balanced, indicating that language difficulty was probably the main reason for the characteristically unbalanced cognitive profile.

In the higher level-development group’s results for the practical reasoning subscale (FQ > 70), there was also no obvious unbalance in respect to the six GMDS fields–a finding that may be related to fact that there was found to be mild or no language difficulty in the children of this group, because the practical reasoning subscale incorporates mathematical concepts as well as ethics and moral issues requiring higher language comprehension ability. Clinically, children in these two groups are more likely to be described as having high-functioning ASD, with an average or above-average developmental quotient and no significant ID or language difficulty (Ousley and Cermak, 2014).

Mixed ANOVA analysis indicated a significant difference within the six subscale quotients of the GMDS, further verifying that the cognitive structure of autistic children was not balanced, and is simultaneously affected by the severity of autism, sex and age. Furthermore, age and autism severity had interacting effects on the overall level of development. These findings suggest that, before receiving intervention or education, autistic children need to undergo a standardized developmental assessment to identify their relative strengths and weaknesses, and to facilitate the choice of educational center and the formulation of a personalized intervention plan.

The GQ and six subscale quotients recorded for the group of children with more severe autism severity were significantly lower than those recorded for the group with less severe levels of ASD. Across the whole group, the GQ and six subscale quotients were negatively correlated with autism severity, suggesting that developmental level is closely correlated with symptom severity in autistic children. Among these, the personal–social and language domains showed a higher correlation with ASD severity, reflecting the close association between these two domains and the core symptoms of ASD. Early developmental levels, particularly the developmental quotient pertaining to the performance subscale, could predict later childhood IQ levels (Sutcliffe et al., 2010), and the results of our study would appear to further verify the hypothesis that autism severity increases with decreases of IQ (Mayes and Calhoun, 2011). These findings also suggest that ASD and its common comorbidity ID may overlap in pathogenesis (Coll-Tané et al., 2019). A lower IQ and the more severe social–communicative features of ASD are associated with lower adaptive functioning in the future (Tillmann et al., 2019), and so it is essential that interventions are developed to improve adaptive skills across different developmental levels and ASD severity. In addition, our study found that the age at diagnosis, for children with more severe ASD, was significantly lower than that of children in the less severe group, and the age was negatively correlated with ASD severity in the whole group. This reflects a common clinical phenomenon that children with more severe ASD symptoms often come to the hospital earlier for evaluation and diagnosis than children with less ASD severity.

In terms of prevalence, ASD has been established to be one of the neurodevelopmental disorders that is different according to sex (Mahendiran et al., 2019), a finding that is also reflected in this study, with a boy-to-girl ratio of 5.5:1. Although there was no significant sex difference in terms of age at diagnosis or autism severity, girls were recorded as having significantly lower scores in the GQ, eye–hand coordination, performance, and practical reasoning GMDS subscales than boys, and the proportion of girls with significant developmental delays in the performance subscale was higher, indicating sex differences in the developmental levels of autistic children. Moreover, boys with ASD may have better visuospatial skills than girls with ASD, since the performance subscale mainly measures visual perception abilities. Bölte et al. (2011) studied sex differences in relation to cognitive domains in 35 males and 21 females described as having higher-functioning ASD, and found that visual attention to detail in males with ASD was superior to that for girls, and proposed that this might be a potential basis for specific cognitive strengths in males with ASD, such as scientific or technical skills. Matheis et al. (2019) assessed the developmental functioning of 1,317 children with ASD aged 17–37 months through reference to the Battelle Developmental Inventory, and their results showed that females with ASD had greater motor difficulties and less communication challenges compared to males. The present study found that, although eye–hand coordination (fine motor) difficulties were more severe in girls, there were no sex differences in gross motor skills, personal social skills, or language skills. However, Duvall et al. (2020) research concluded that there were no sex differences concerning the cognitive abilities of young children with ASD aged 18–68 months. These different results may relate to the differences in sample sizes, sample ages, and test tools, which need to be further explored.

The distribution of the subscale quotients of the GMDS across different age groups suggested that locomotor skills tend to decrease in line with age at diagnosis. This pattern is consistent with findings from previous studies that motor difficulties become more pronounced with age (Landa and Garrett-Mayer, 2006; Lloyd et al., 2013; Licari et al., 2020). The transition from infancy to preschool child requires the acquisition of increasingly complex movement skills through increases in muscle strength, coordination, and stability (Licari et al., 2020). If a child has challenges in acquiring simple movement skills, it will be more difficult to acquire complex movement skills during subsequent development stages. This may account for the relatively poorer locomotor quotients corresponding with an increasing age in the present study. Some prospective follow-up studies of high-risk infants across early development have found that motor difficulties in the infancy period is associated with later ASD diagnosis or ASD symptoms (Estes et al., 2015; Paquet et al., 2016; West, 2019). Therefore, motor difficulties may be an early marker preceding a diagnosis of ASD, but longitudinal follow-up studies are needed for further verification.

Although language skills and eye–hand coordination varied among the age groups in our study, they did not decline with age. Interestingly, in the language domain, the group comprising children aged 18–24 months had the lowest CQ scores, and the 36–48 months group had the highest CQ scores, with no significant fluctuation after 48 months. This could be explained by the fact that most children with ASD do not have meaningful language skills until 24 months old, and 24–48 months is a rapid period of language development in children with ASD. In turn, this might indicate that the level of language development at 48 months may predict the language prognosis in ASD. Brignell et al. (2018) found that language ability at 4 years and IQ rather than social communication skills influence the language prognosis in children with ASD. However, a longitudinal follow-up study is needed to verify this deduction. In respect of the eye–hand coordination subscale, the 18–24 months group recorded the highest DQ scores, with no significant fluctuation after 24 months. It may be that the test items in this domain before the age of 24 months are mainly based on perceptual observation, which does not require a high level of language comprehension. However, after the age of 2 years old, the need for language comprehension in this area is increased. Hence, the DQ scores of the later age group were lower than those of the 18–24 months age group.

In-depth exploration of the dataset using multi-way ANOVA demonstrated that autism severity and age had major effects on almost all GMDS subscales and GQ, indicating that children with ASD at different ages and with different levels of autism severity had different developmental levels in various areas of cognitive structure. In addition, sex had a major effect on performance quotient, further indicating that boys with ASD may perform better than girls in this domain, which mainly measures visual perception ability. To date, this is the first report in China on the effects of autism severity, sex, and age on the different cognitive structure domains measured using the GMDS. The present study contributes to describing the cognitive developmental profiles of children with ASD.

This study has several limitations, including the use of a cross-sectional research design. Although the developmental levels of ASD for different age groups were compared in the present research, the data obtained cannot be taken to represent the development trends of the same groups according to age. In addition, the developmental level of children with ASD at different ages was also affected by the context, drug or rehabilitation therapies and education. Therefore, longitudinal follow-up studies are needed to further verify the effect of age on the developmental level. For the comparison of sex differences in relation to developmental level, no typically developing children were included as a control group, and the ratio of boys to girls in this study was 5.5 to 1. Although some cognitive differences between boys and girls were detected, they are likely to be affected by this sex unbalance, which is another limitation of the study; however, given the identified sex and age differences in the relative development levels of autistic children, determining the most effective means with which to make up for these deficiencies, as well as how and when to select and provide the most appropriate interventions, will be an important future extension of our research in the future.

## Data Availability Statement

The raw data supporting the conclusions of this article will be made available by the authors, without undue reservation.

## Ethics Statement

The studies involving human participants were reviewed and approved by the Ethics Committee of the First Hospital of Jilin University. Written informed consent to participate in this study was provided by the participants’ legal guardian/next of kin.

## Author Contributions

H-HL and F-YJ participated in the design and definition of this study. C-XW, J-YF, and C-LL provided assistance for data acquisition and literature search. H-HL and C-XW performed the statistical analysis and drafted the manuscript. BW and J-YF carried out the manuscript editing. All authors have read and approved the content of the manuscript.

## Conflict of Interest

The authors declare that the research was conducted in the absence of any commercial or financial relationships that could be construed as a potential conflict of interest.

## Abbreviations

ASD: autism spectrum disorder
IDs: intellectual disabilities
ADOS: autism diagnostic observation schedule
GMDS: griffiths mental development scales
DSM-5: diagnostic and statistical manual of mental disorders
ABC: autism behavior checklist
CARS: childhood autism rating scale
GQ: general quotient
AQ: locomotor quotient
BQ: personal-social quotient
CQ: hearing and language quotient
DQ: eye-hand coordination quotient
EQ: performance quotient
FQ: practical reasoning quotient.
