# Supplementary figures and images for: A Developmental Profile of Children With Autism Spectrum Disorder in China Using the Griffiths Mental Development Scales
Source: Front Psychol. 2020 Nov 9;11:570923. doi: 10.3389/fpsyg.2020.570923 (PMC7680850; doi:10.3389/fpsyg.2020.570923)

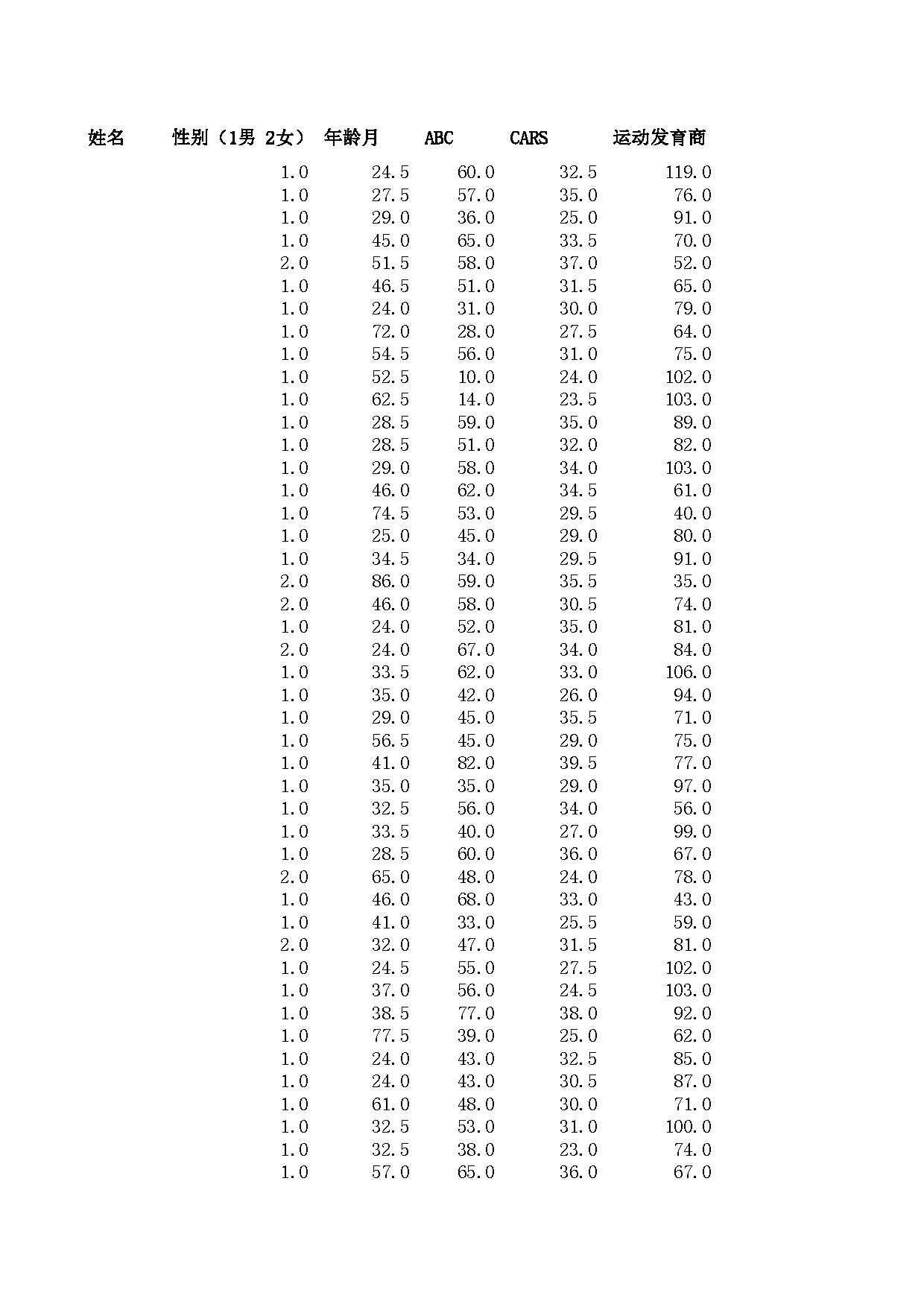

Supplement: Supplementary file 1 [file Data_Sheet_1.zip › Griffiths原始数据-1.jpg]

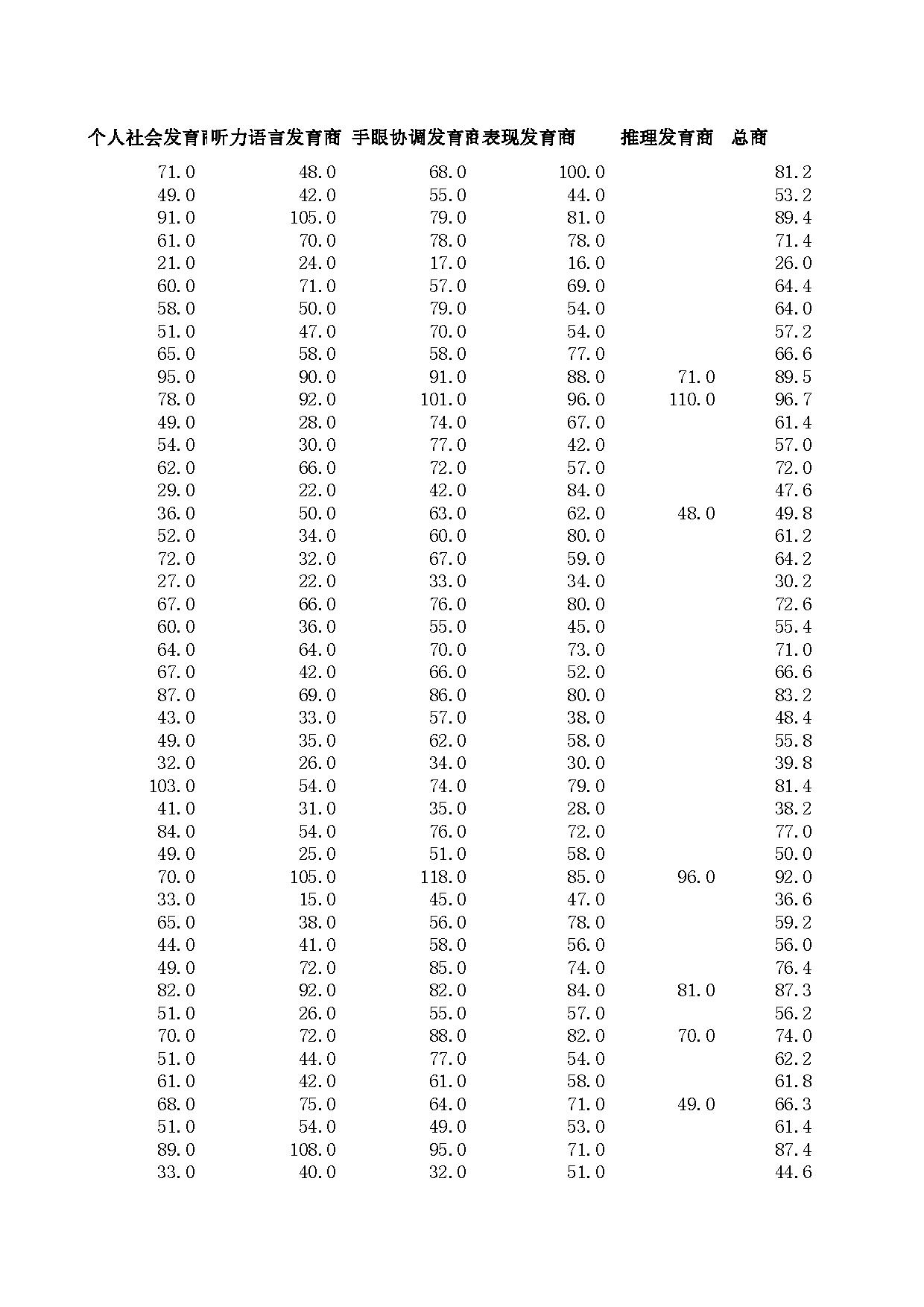

Supplement: Supplementary file 1 [file Data_Sheet_1.zip › Griffiths原始数据-10.jpg]

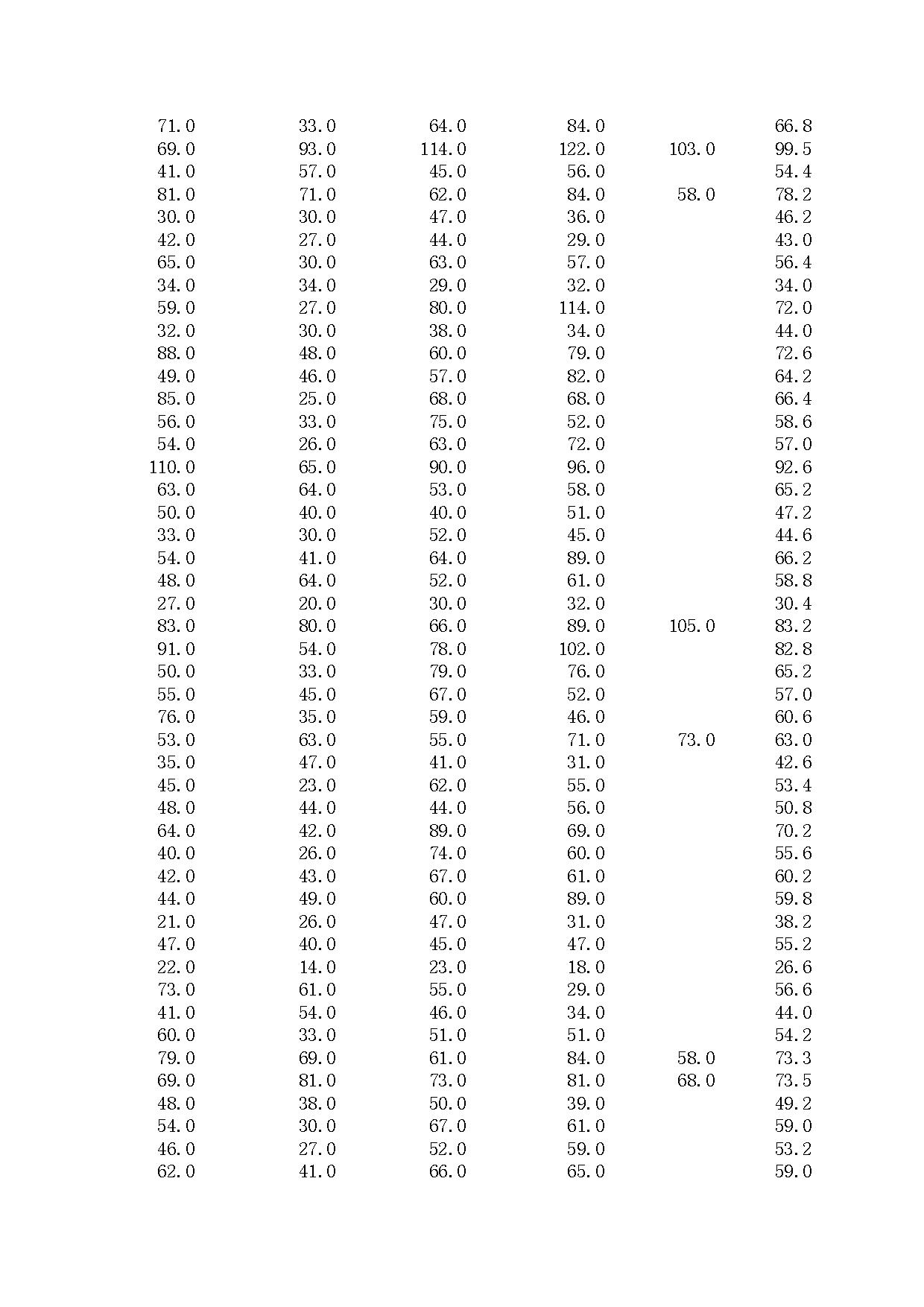

Supplement: Supplementary file 1 [file Data_Sheet_1.zip › Griffiths原始数据-11.jpg]

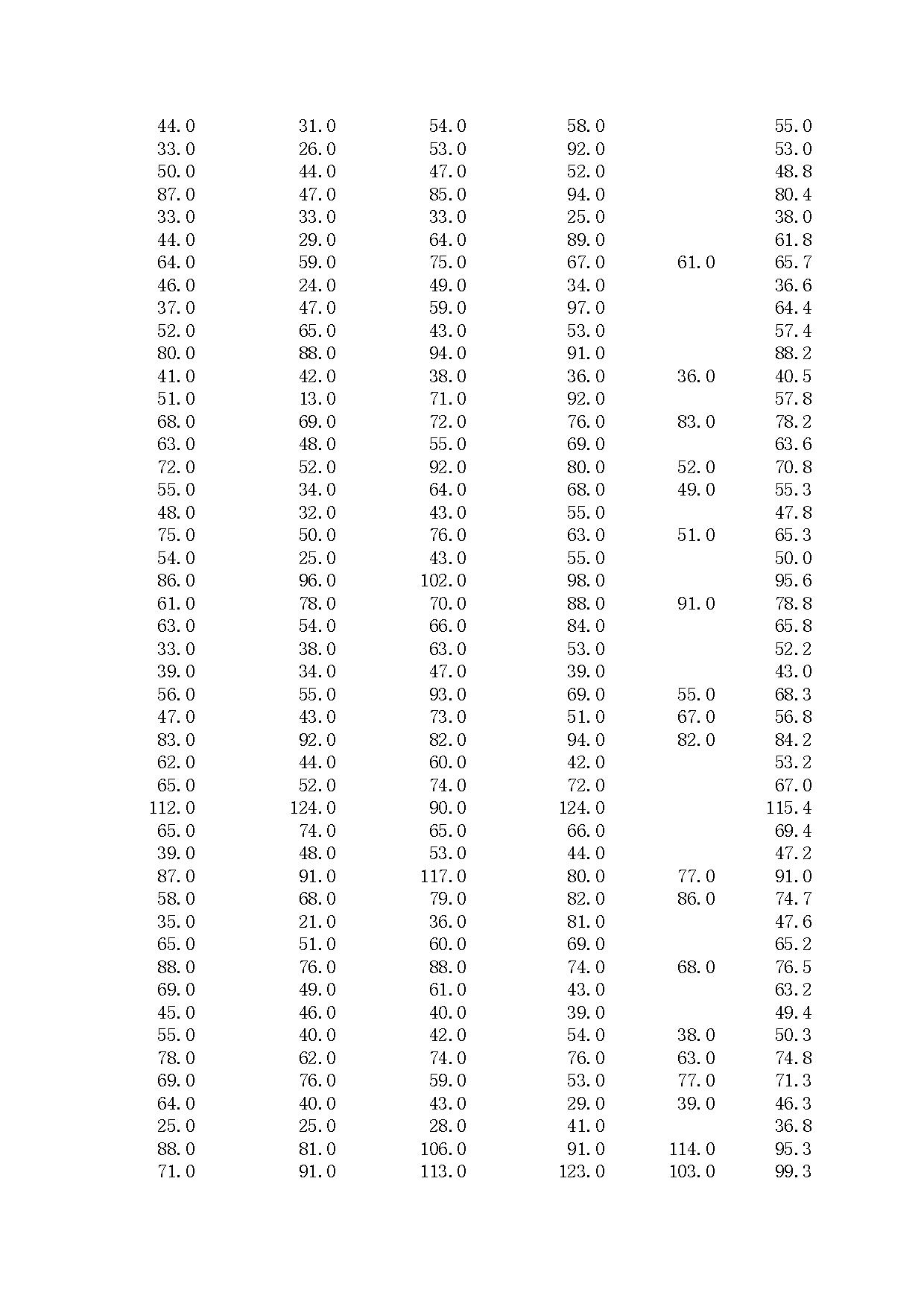

Supplement: Supplementary file 1 [file Data_Sheet_1.zip › Griffiths原始数据-12.jpg]

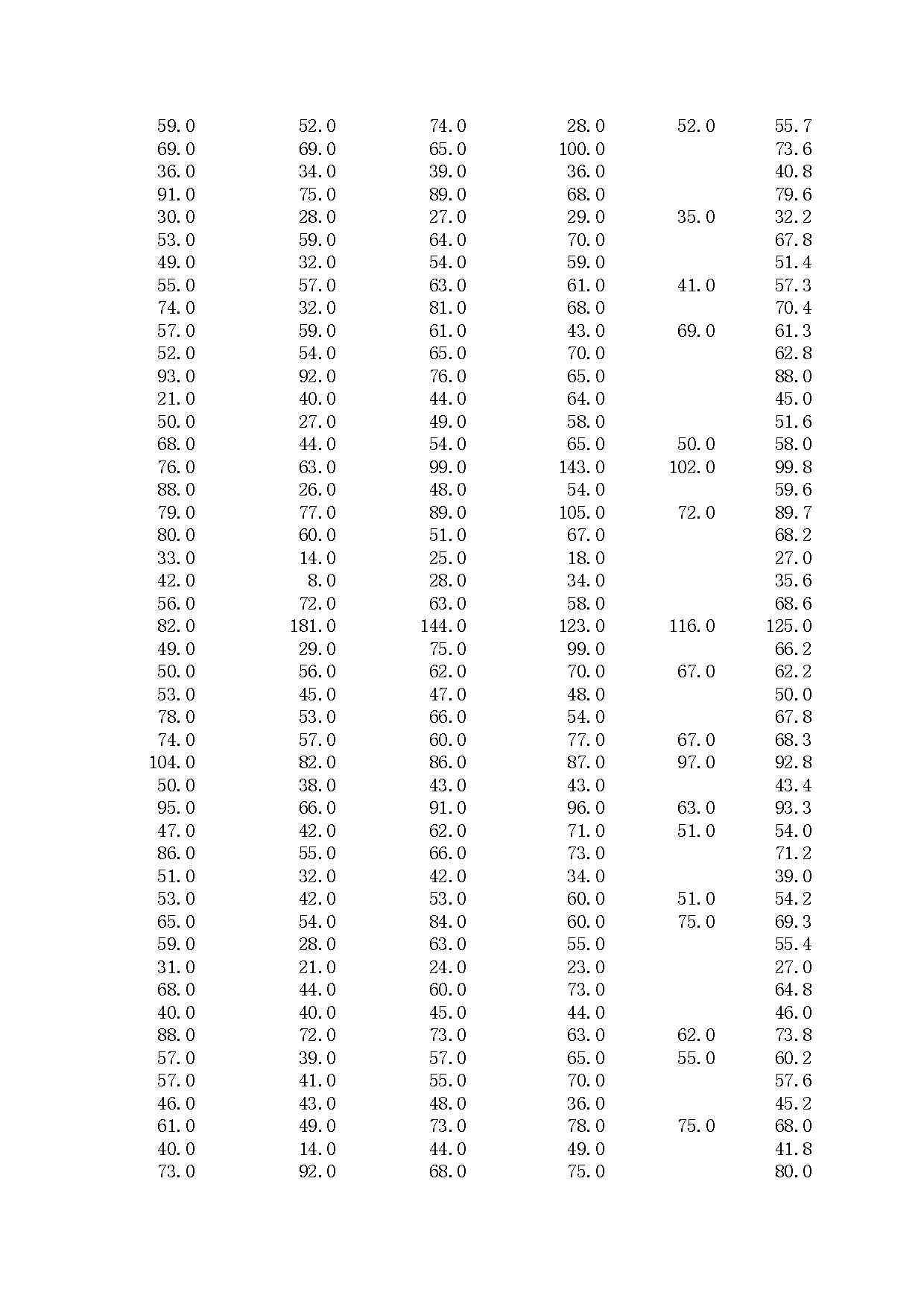

Supplement: Supplementary file 1 [file Data_Sheet_1.zip › Griffiths原始数据-13.jpg]

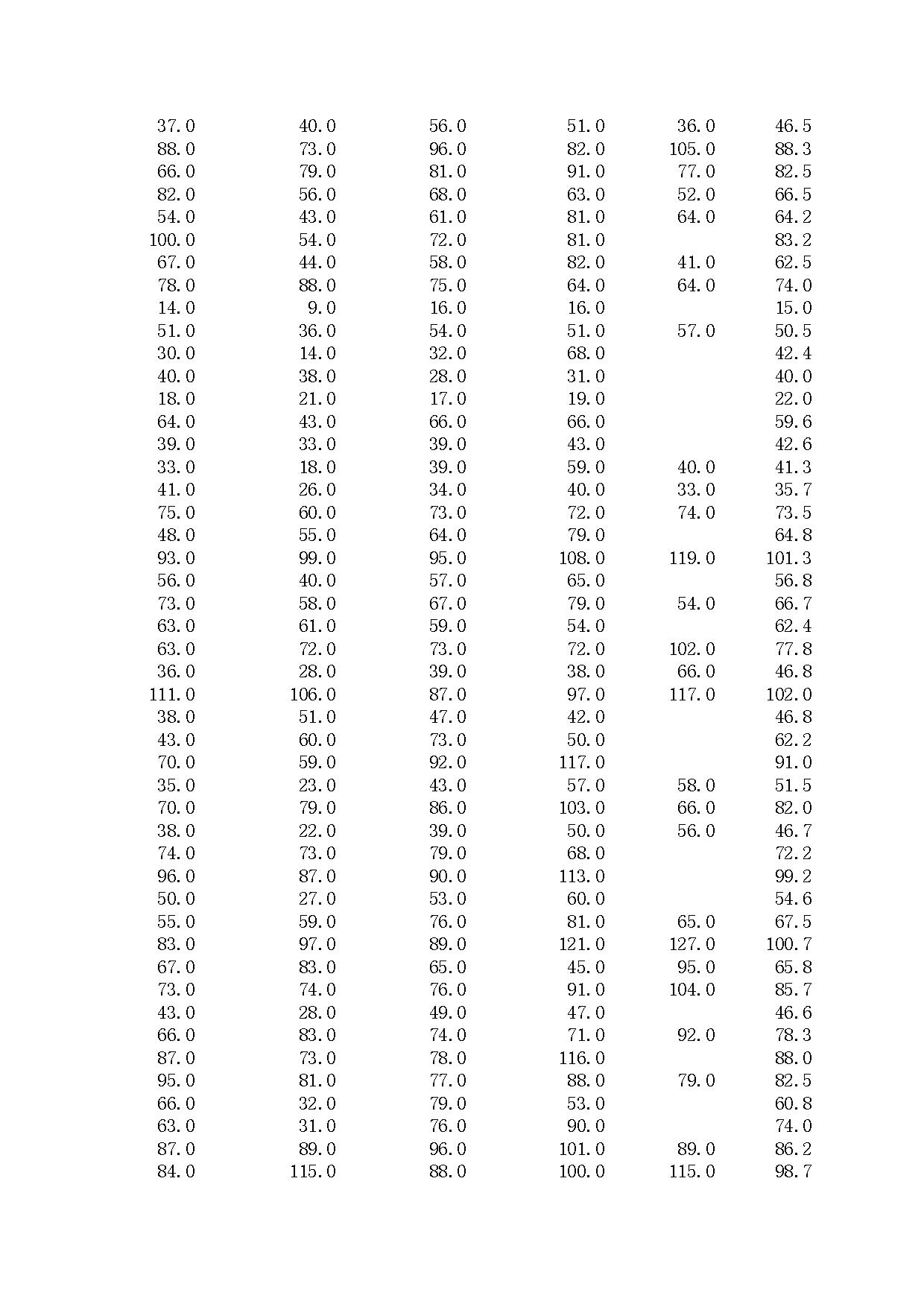

Supplement: Supplementary file 1 [file Data_Sheet_1.zip › Griffiths原始数据-14.jpg]

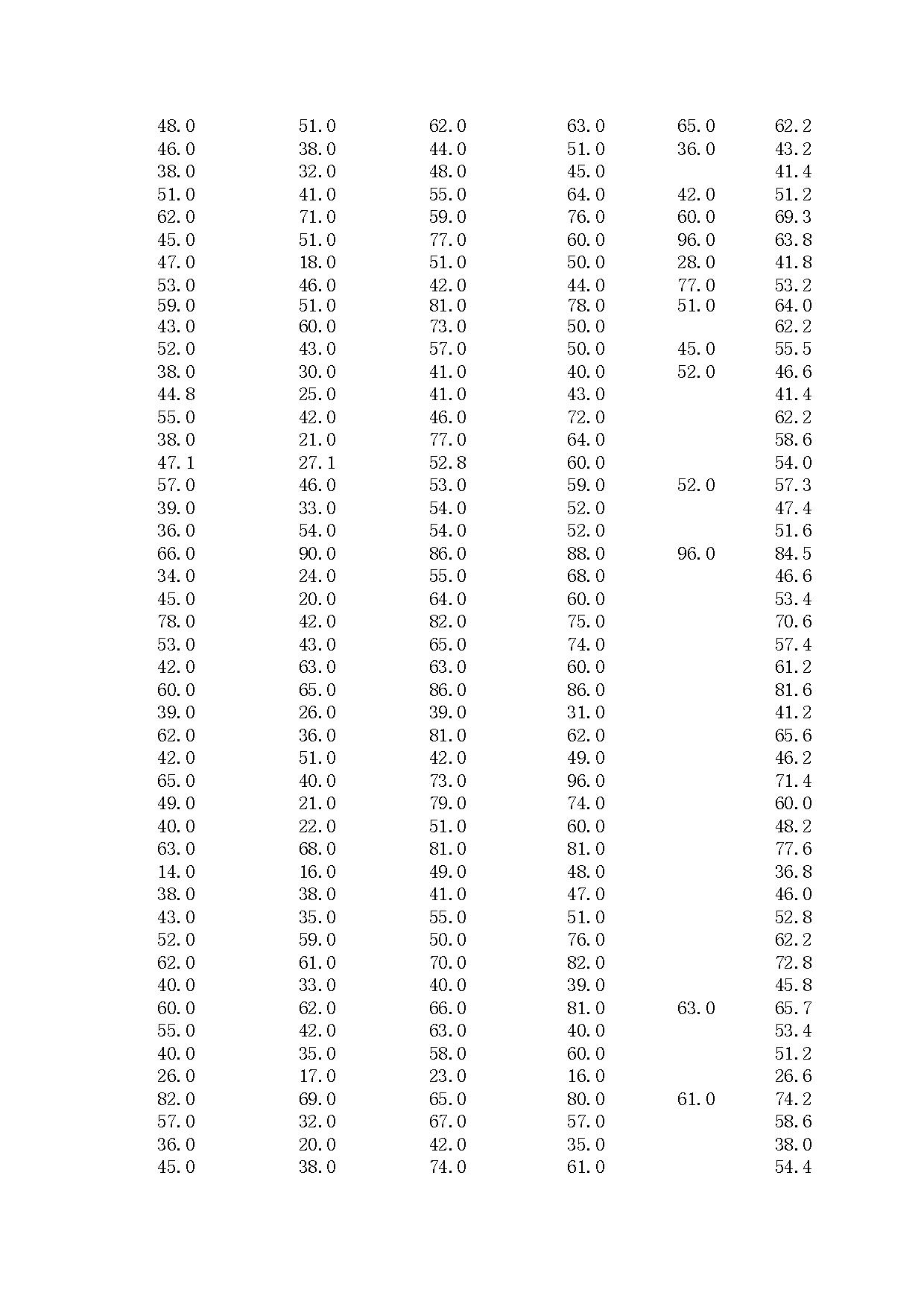

Supplement: Supplementary file 1 [file Data_Sheet_1.zip › Griffiths原始数据-15.jpg]

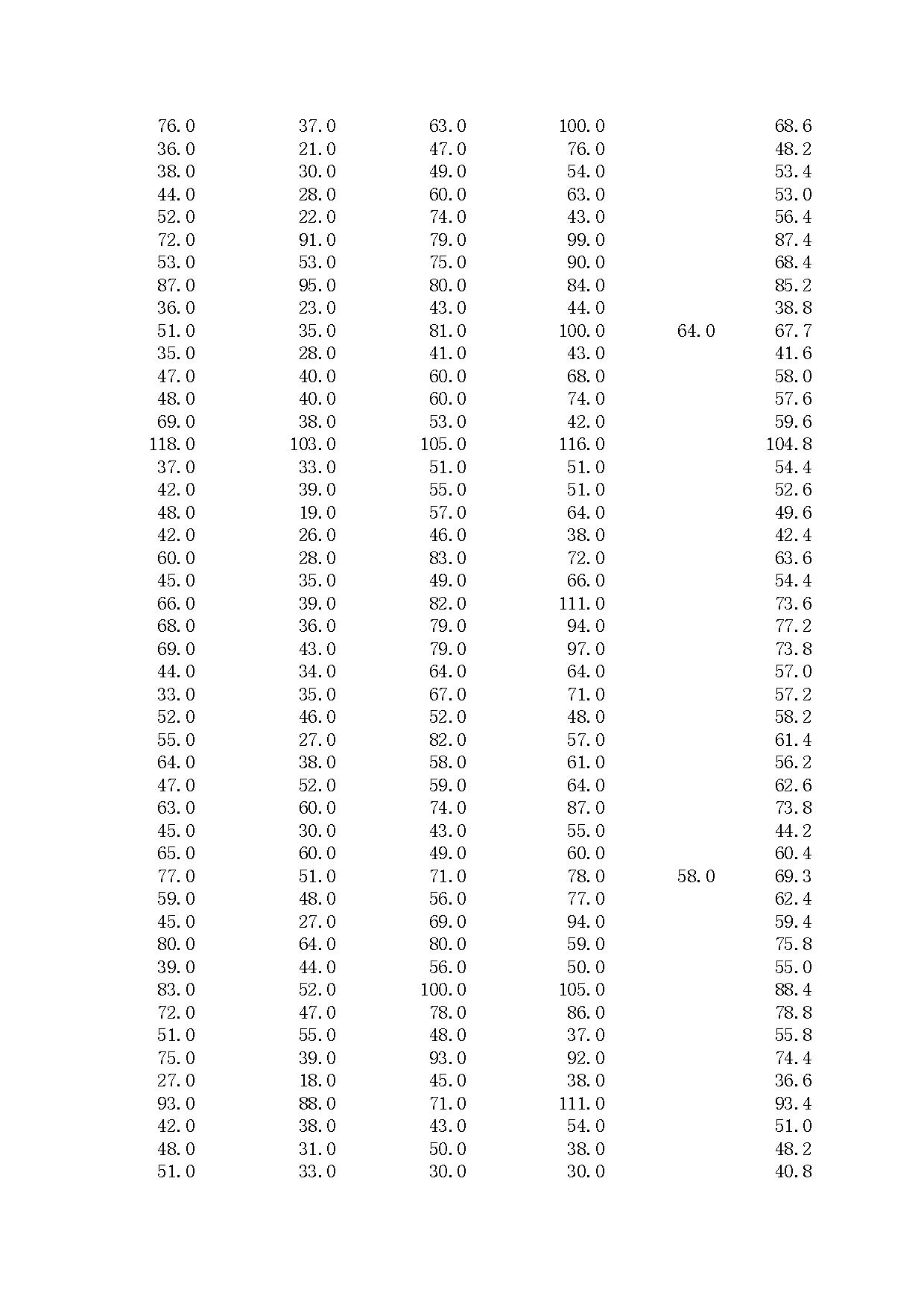

Supplement: Supplementary file 1 [file Data_Sheet_1.zip › Griffiths原始数据-16.jpg]

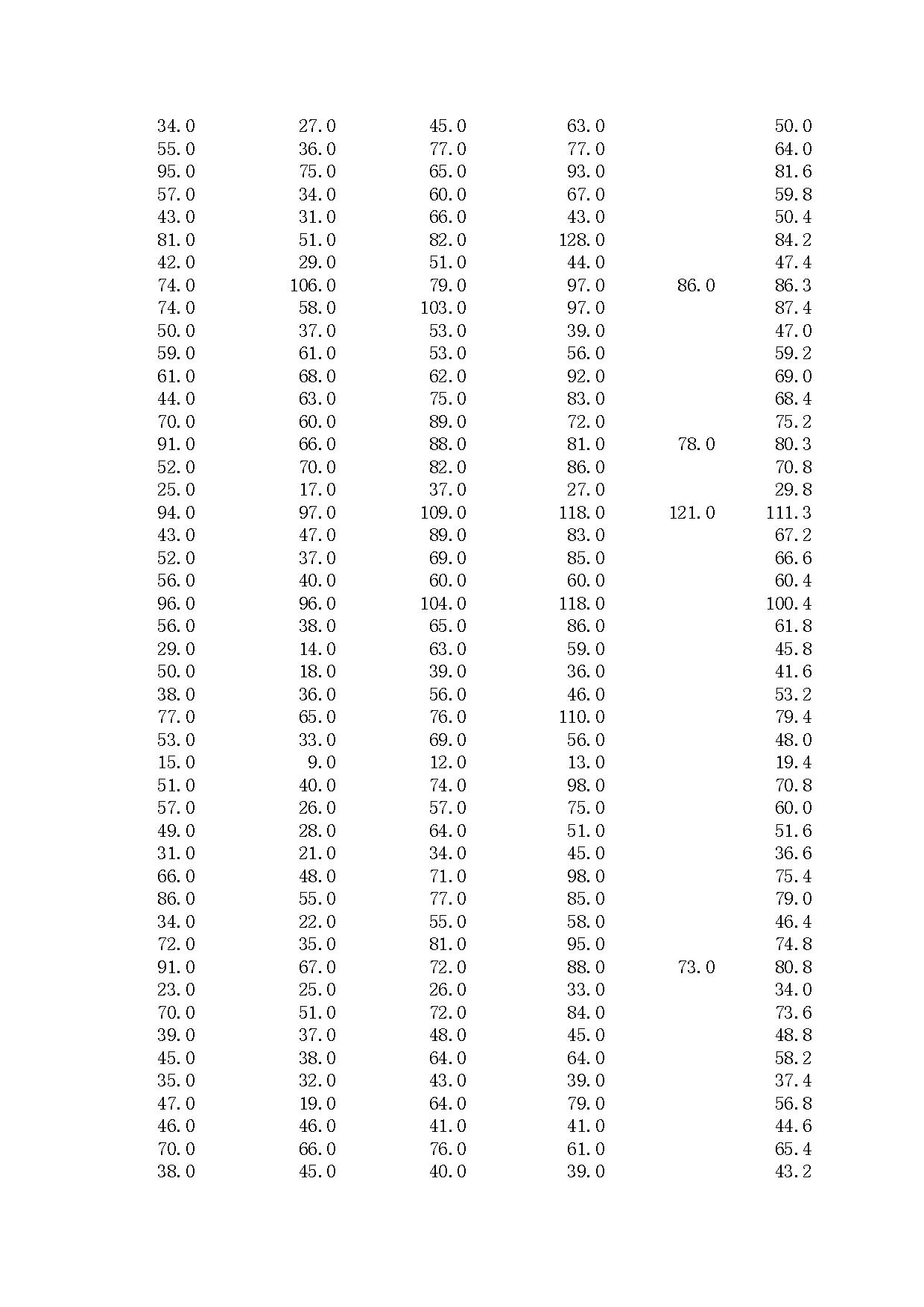

Supplement: Supplementary file 1 [file Data_Sheet_1.zip › Griffiths原始数据-17.jpg]

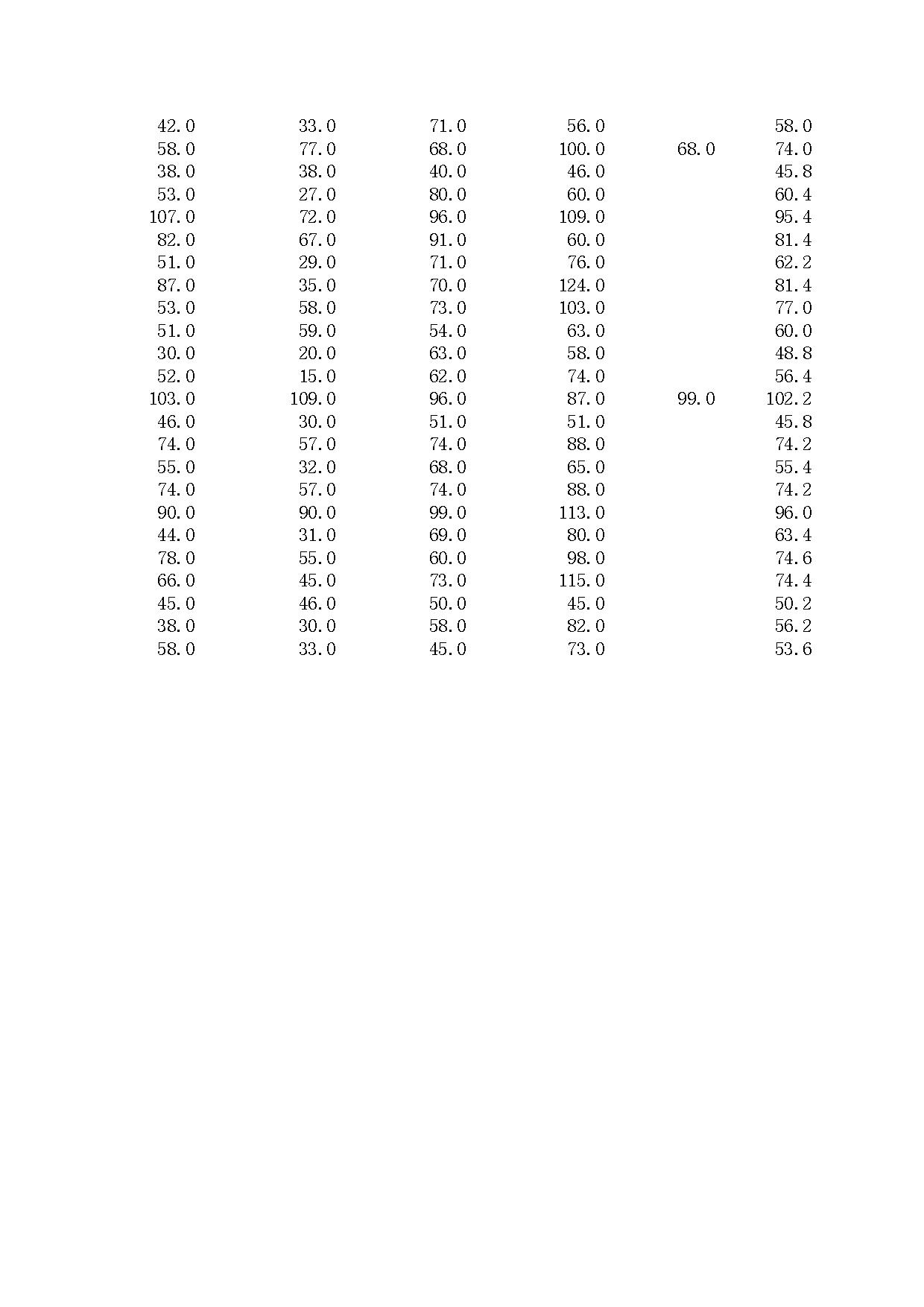

Supplement: Supplementary file 1 [file Data_Sheet_1.zip › Griffiths原始数据-18.jpg]

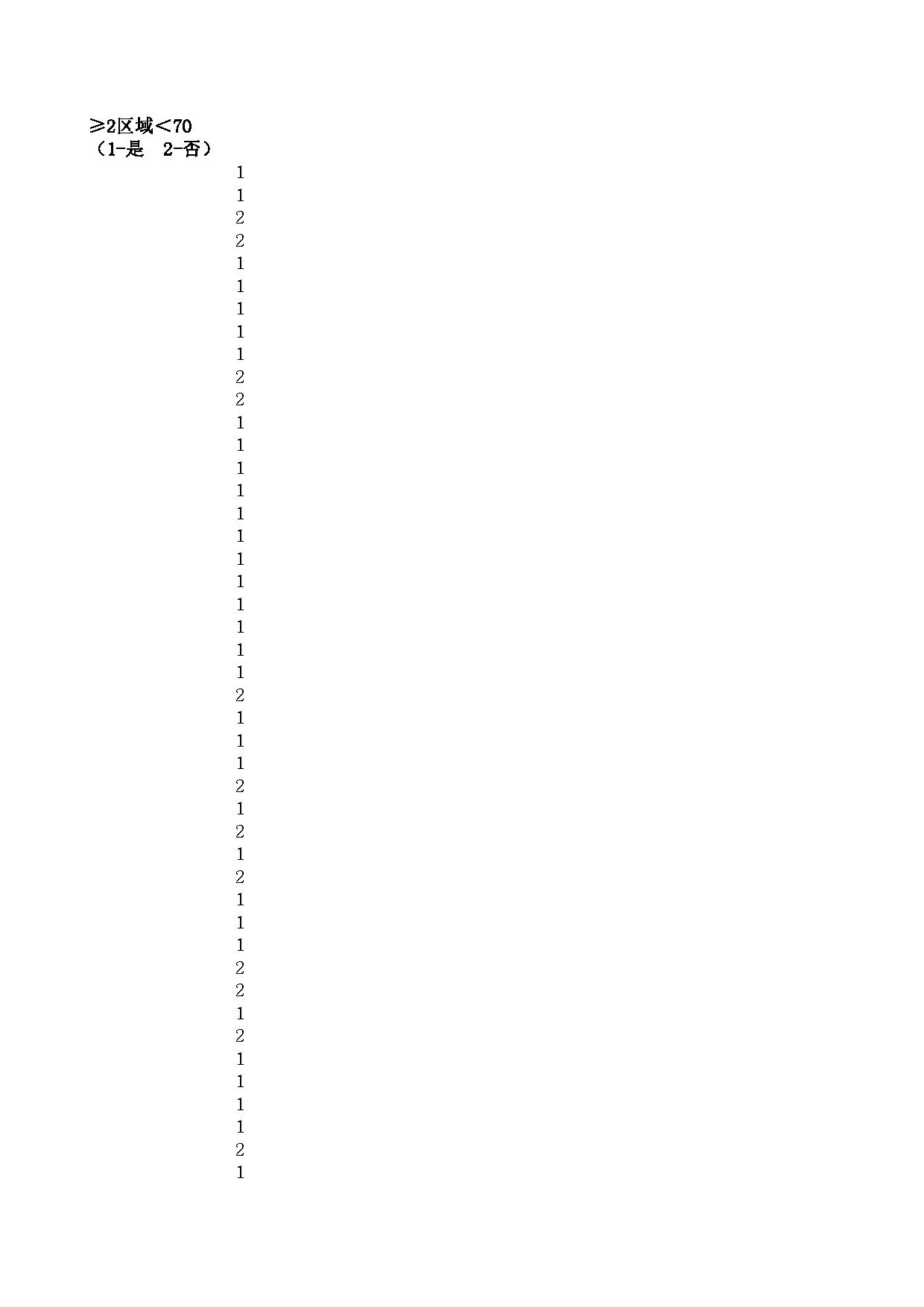

Supplement: Supplementary file 1 [file Data_Sheet_1.zip › Griffiths原始数据-19.jpg]

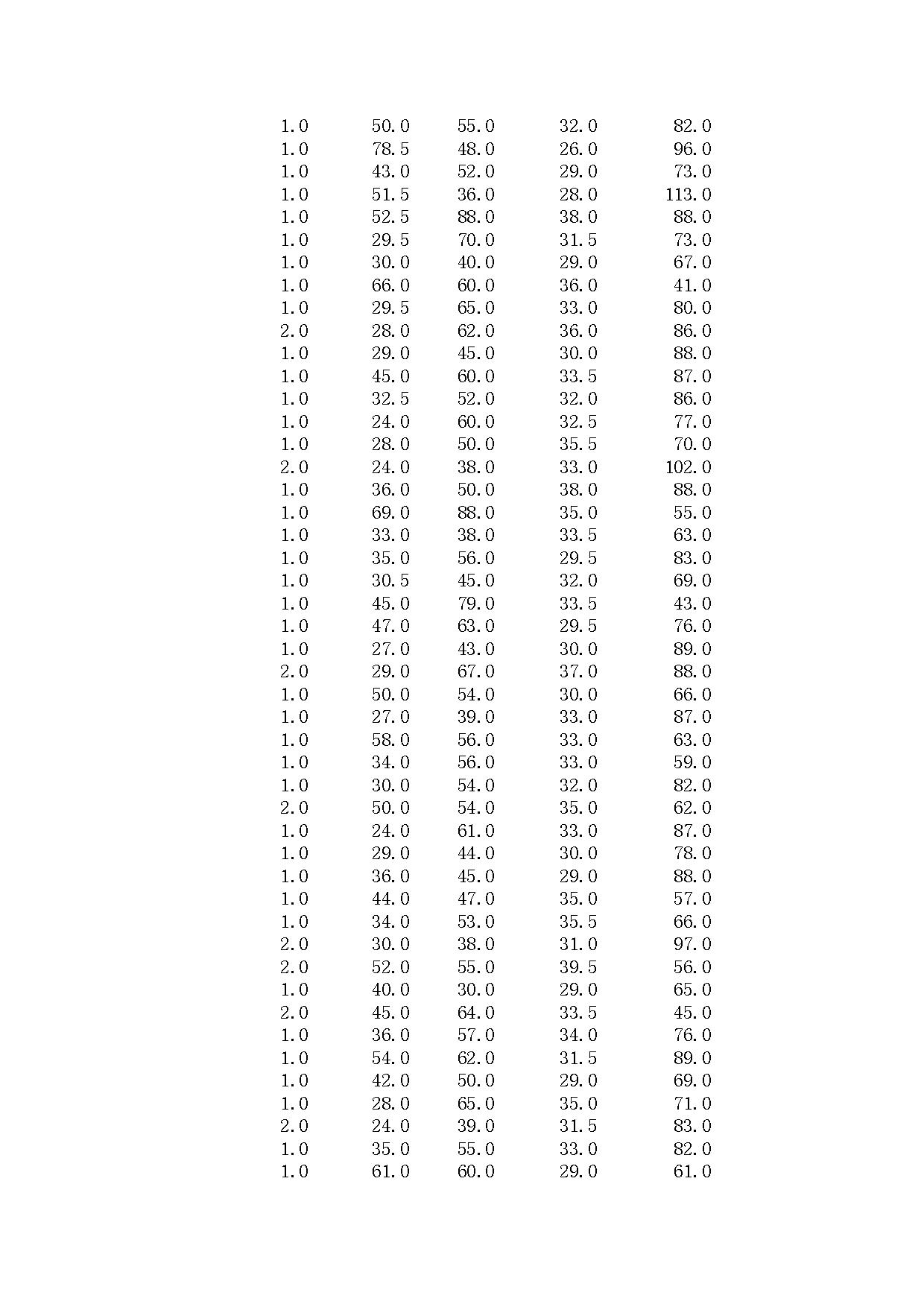

Supplement: Supplementary file 1 [file Data_Sheet_1.zip › Griffiths原始数据-2.jpg]

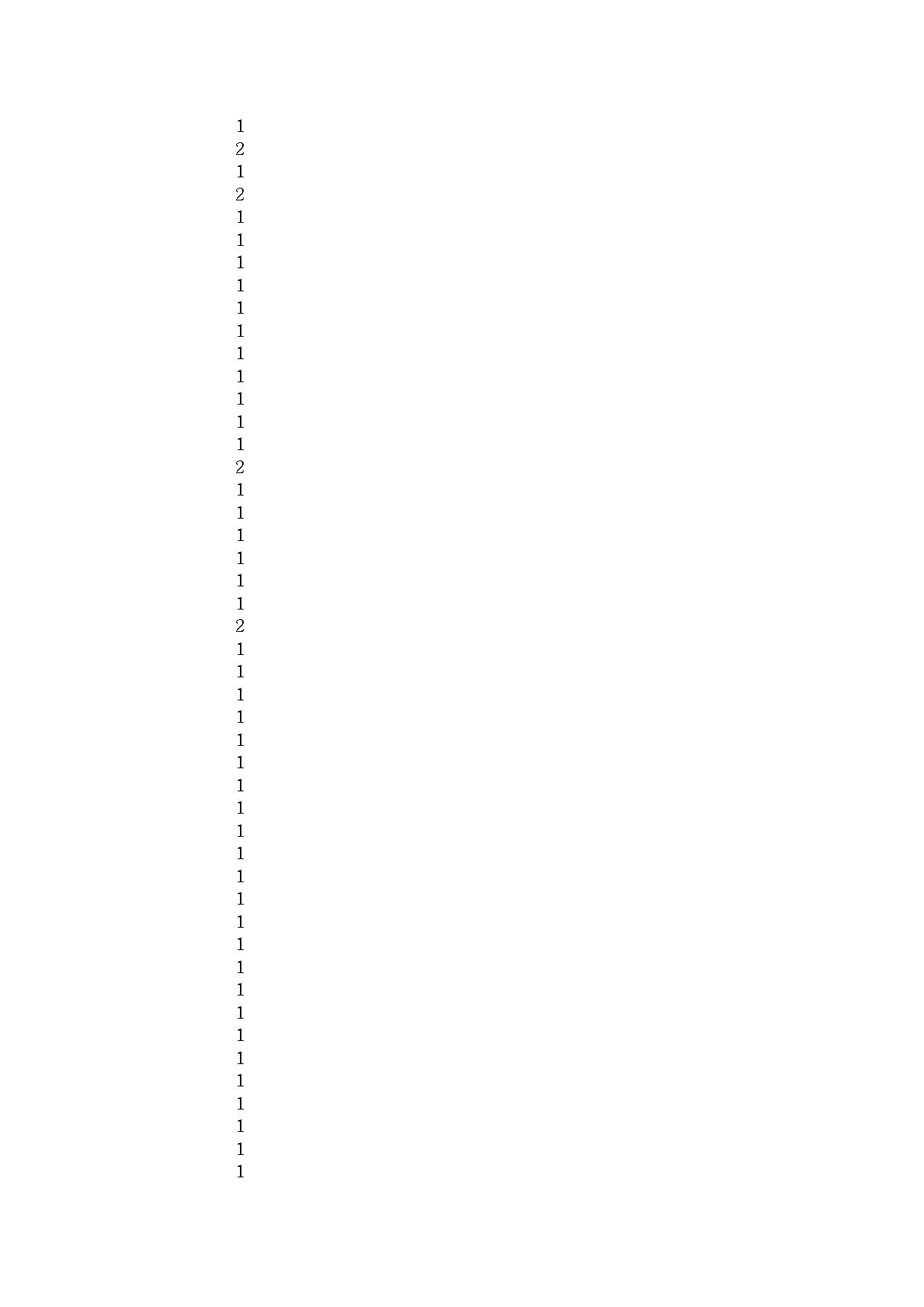

Supplement: Supplementary file 1 [file Data_Sheet_1.zip › Griffiths原始数据-20.jpg]

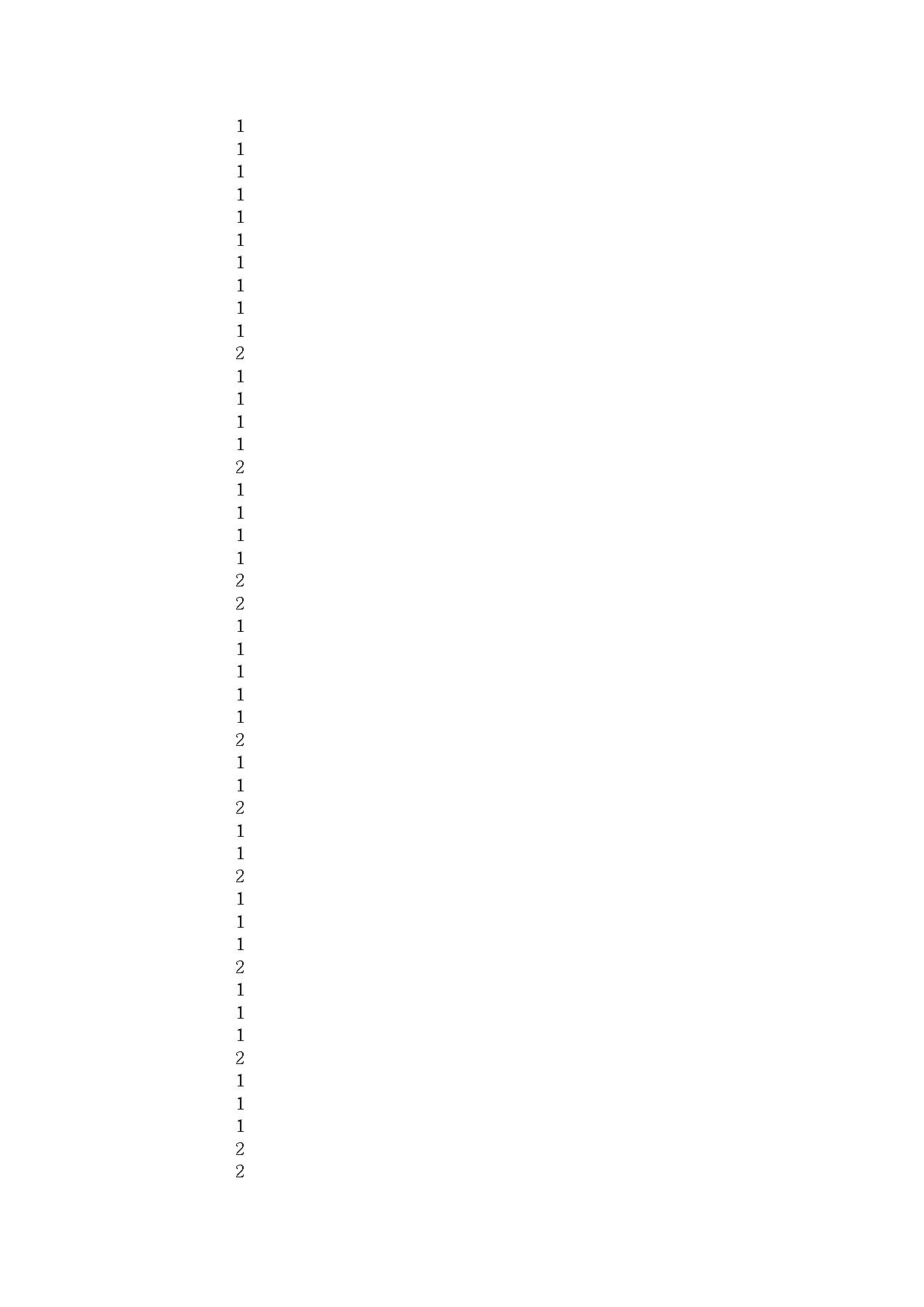

Supplement: Supplementary file 1 [file Data_Sheet_1.zip › Griffiths原始数据-21.jpg]

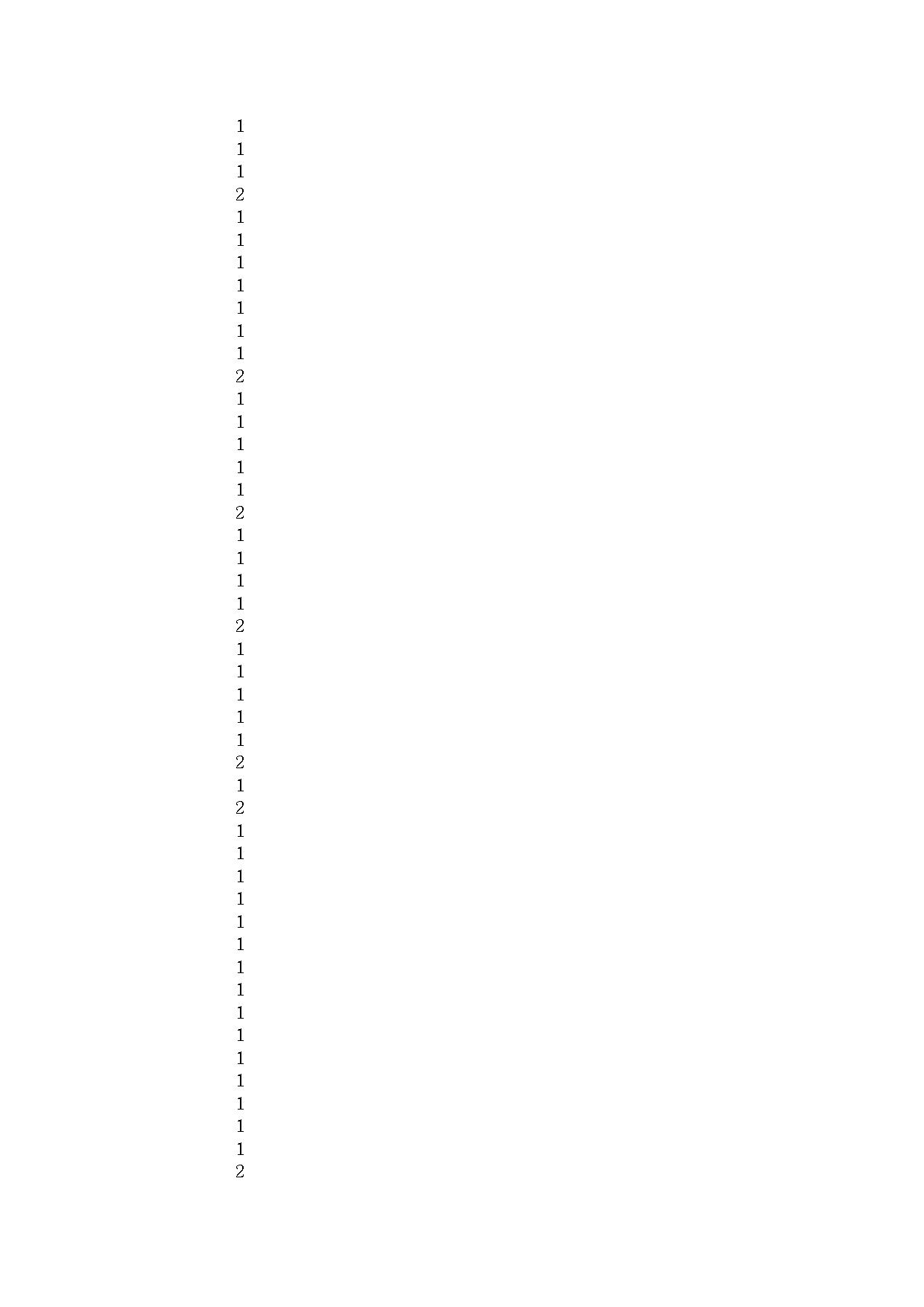

Supplement: Supplementary file 1 [file Data_Sheet_1.zip › Griffiths原始数据-22.jpg]

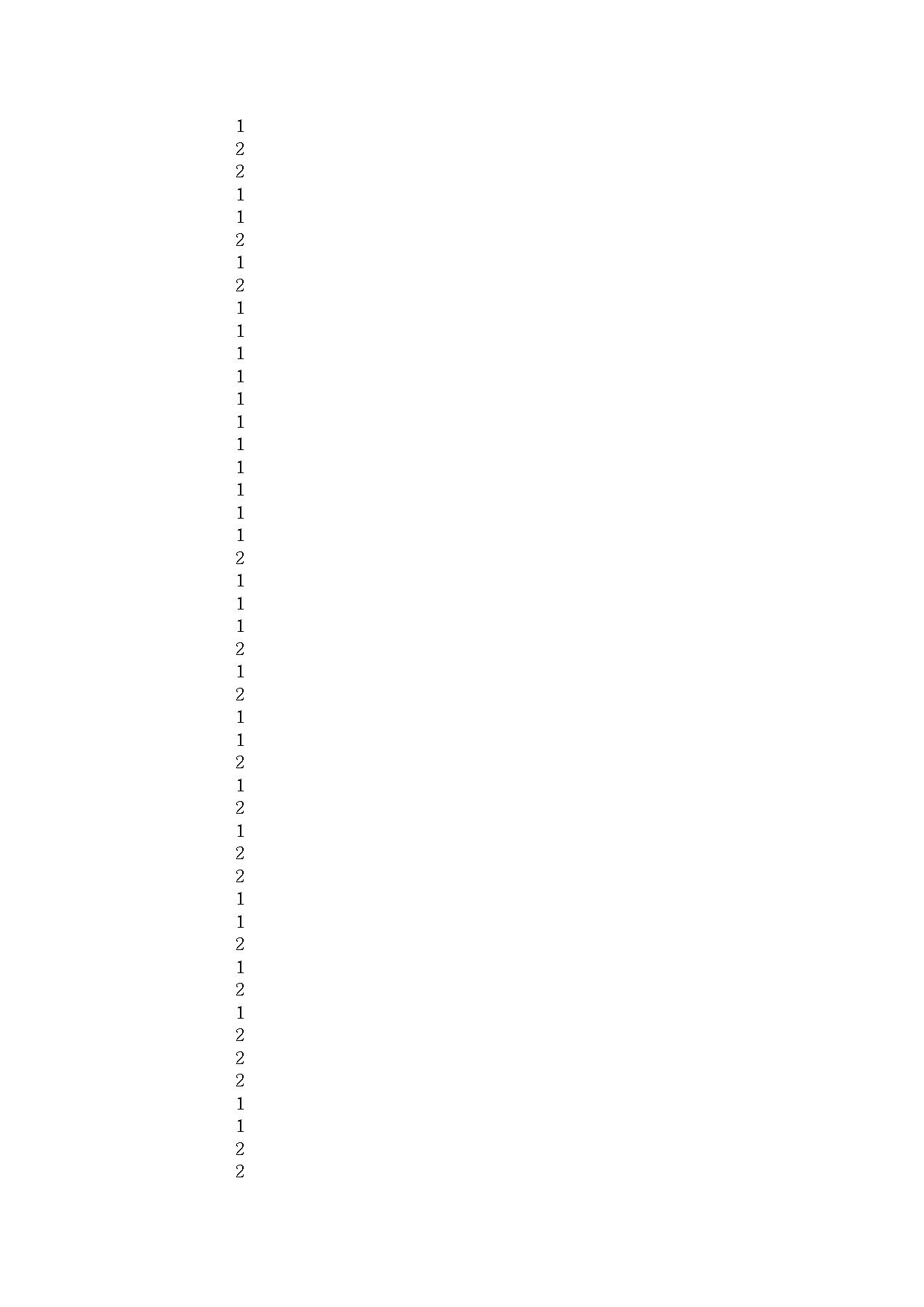

Supplement: Supplementary file 1 [file Data_Sheet_1.zip › Griffiths原始数据-23.jpg]

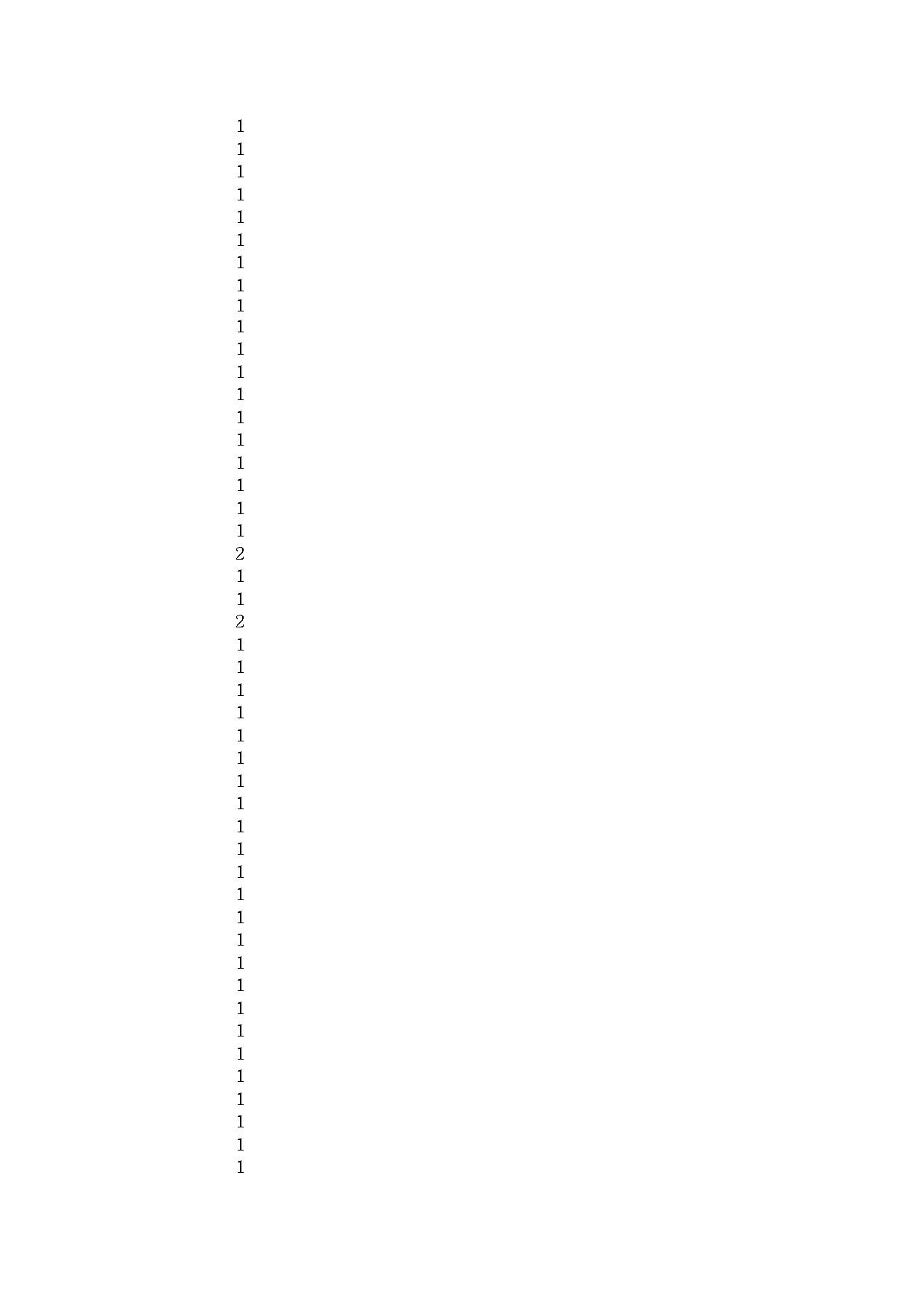

Supplement: Supplementary file 1 [file Data_Sheet_1.zip › Griffiths原始数据-24.jpg]

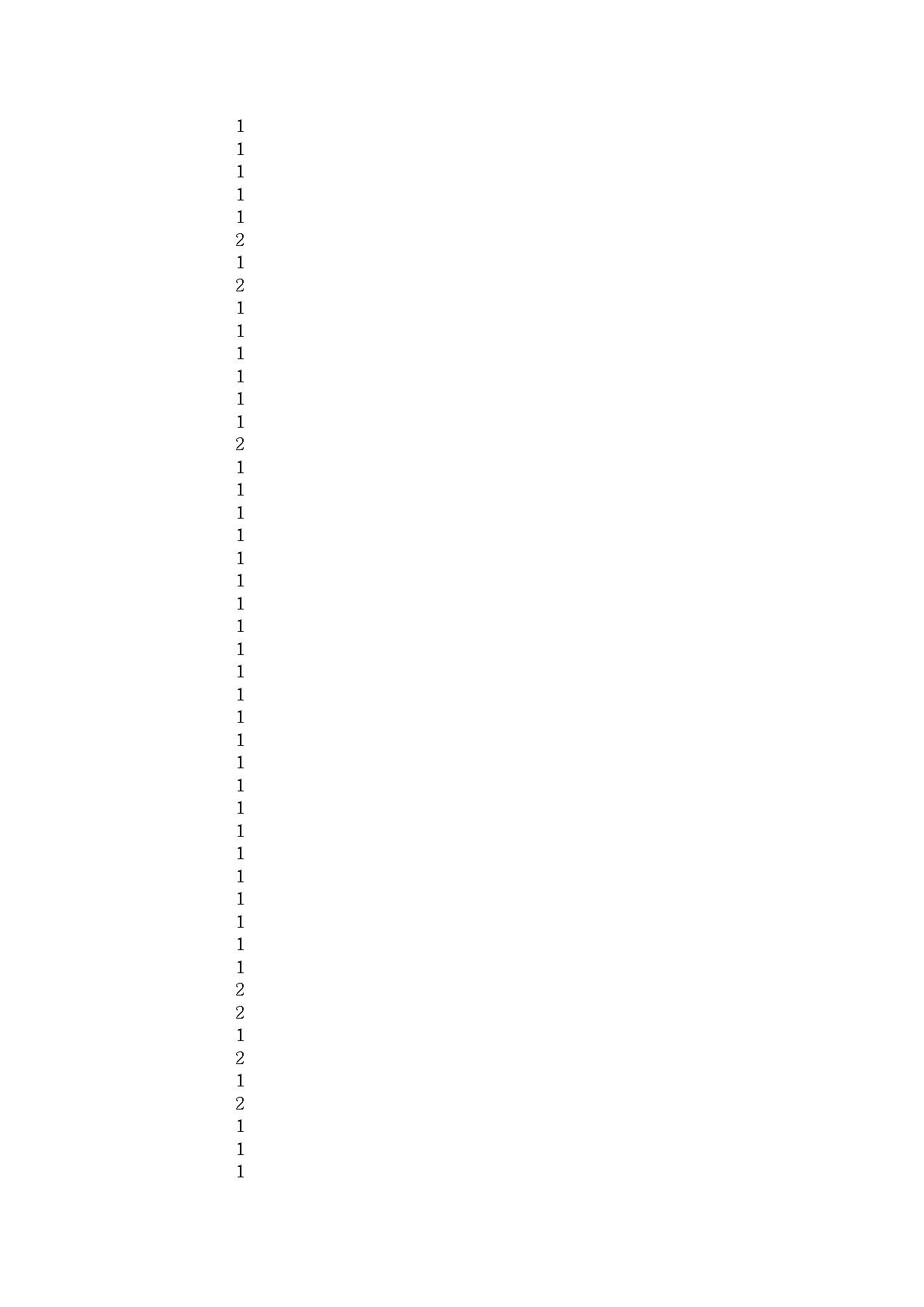

Supplement: Supplementary file 1 [file Data_Sheet_1.zip › Griffiths原始数据-25.jpg]

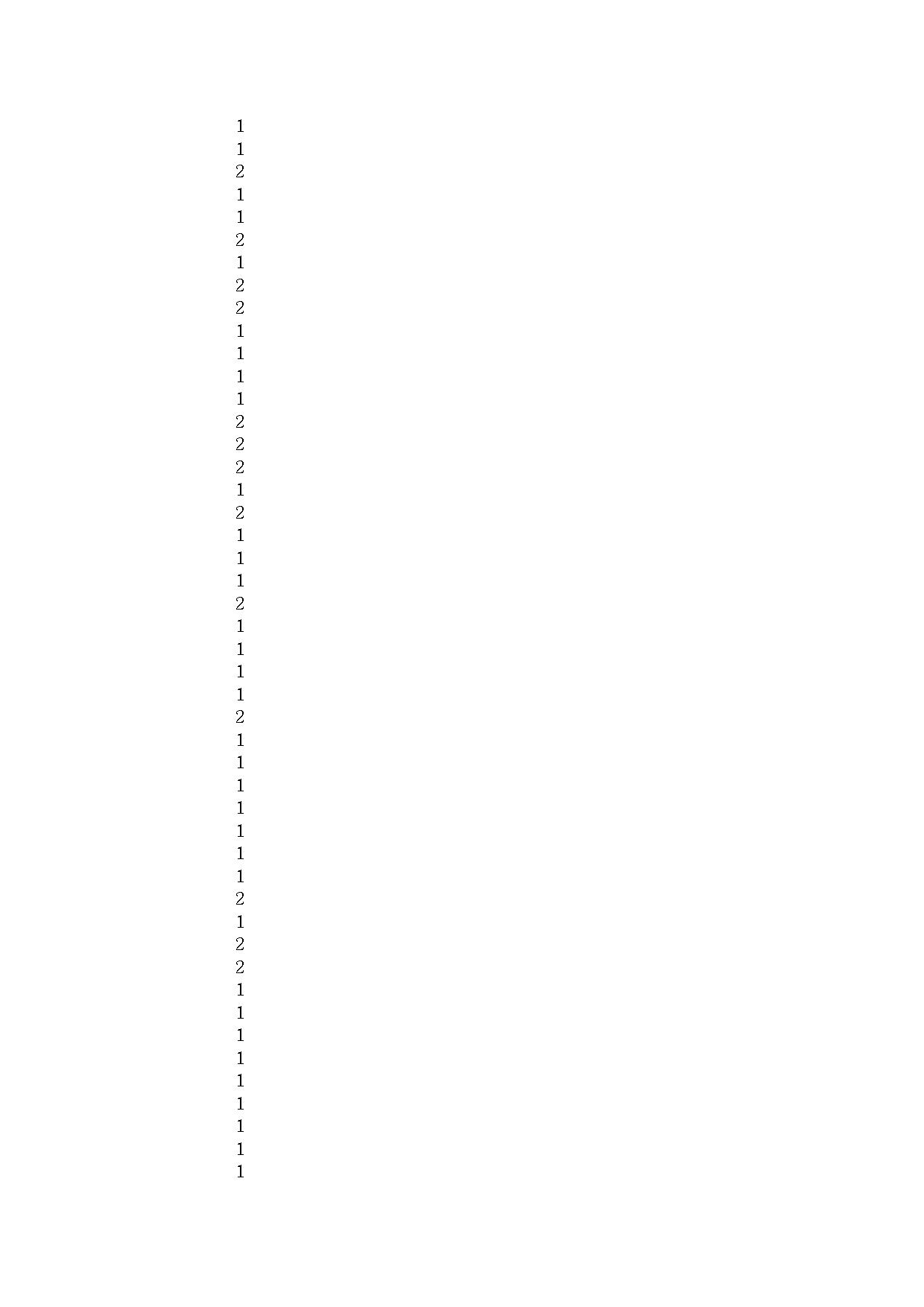

Supplement: Supplementary file 1 [file Data_Sheet_1.zip › Griffiths原始数据-26.jpg]

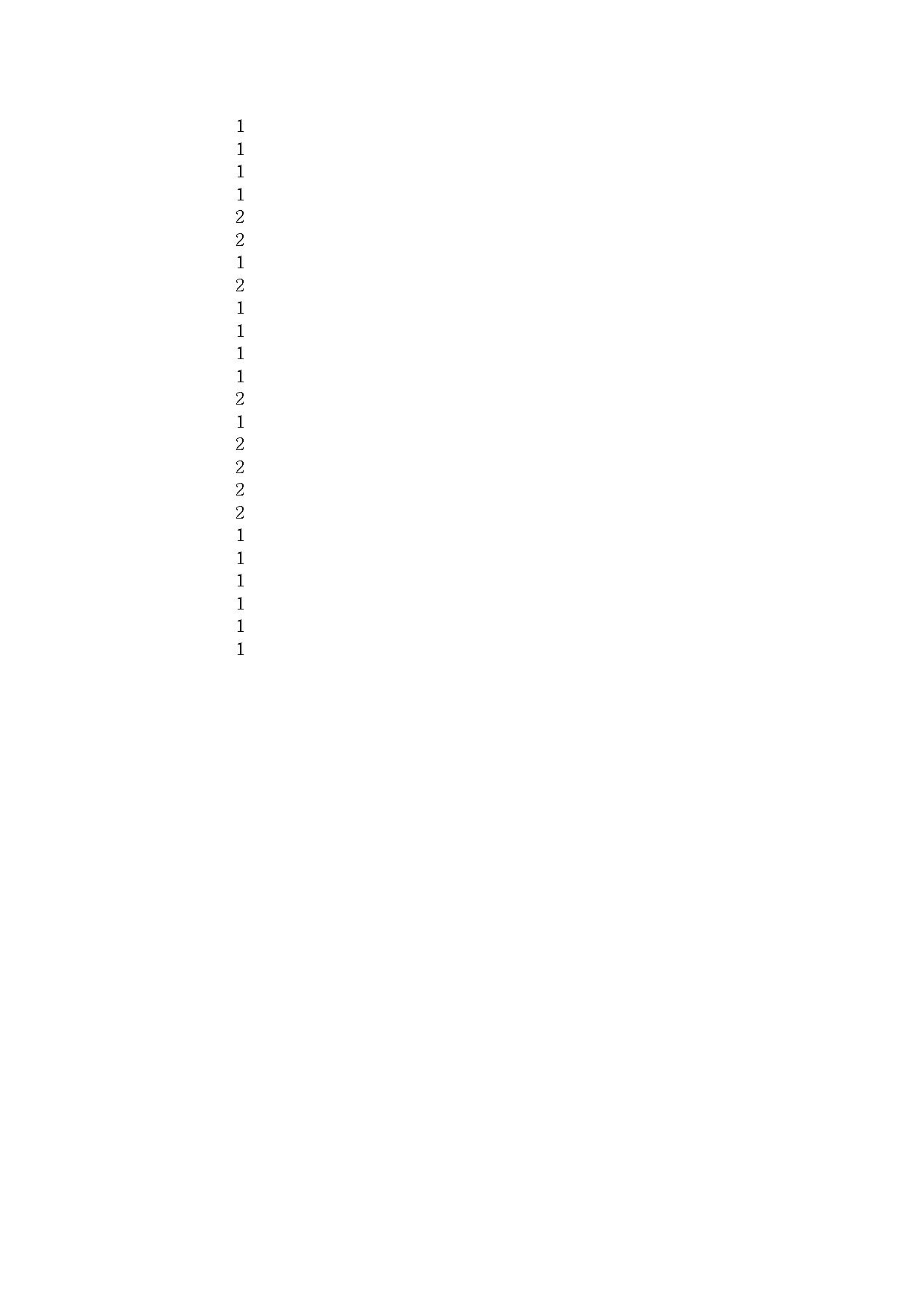

Supplement: Supplementary file 1 [file Data_Sheet_1.zip › Griffiths原始数据-27.jpg]

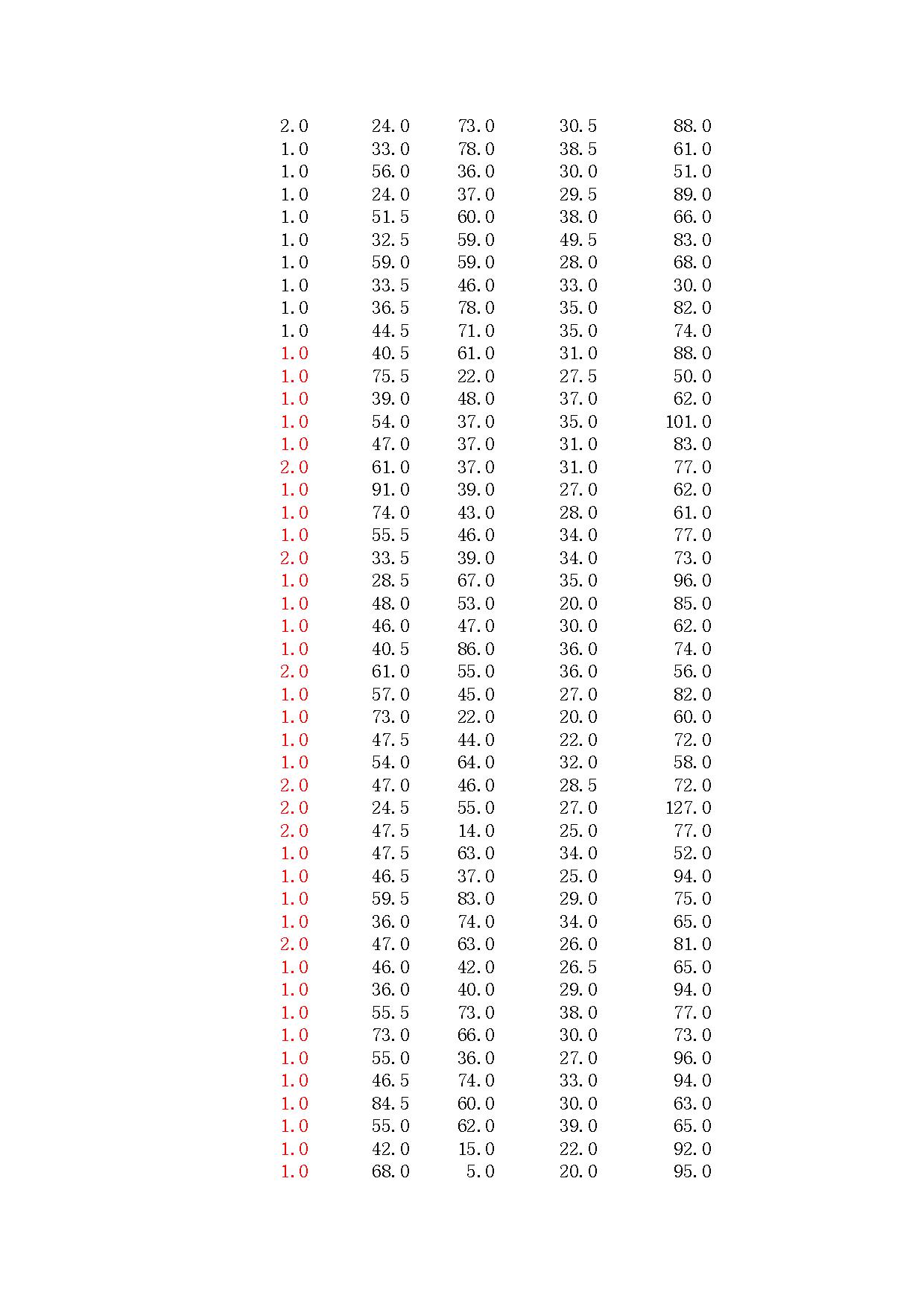

Supplement: Supplementary file 1 [file Data_Sheet_1.zip › Griffiths原始数据-3.jpg]

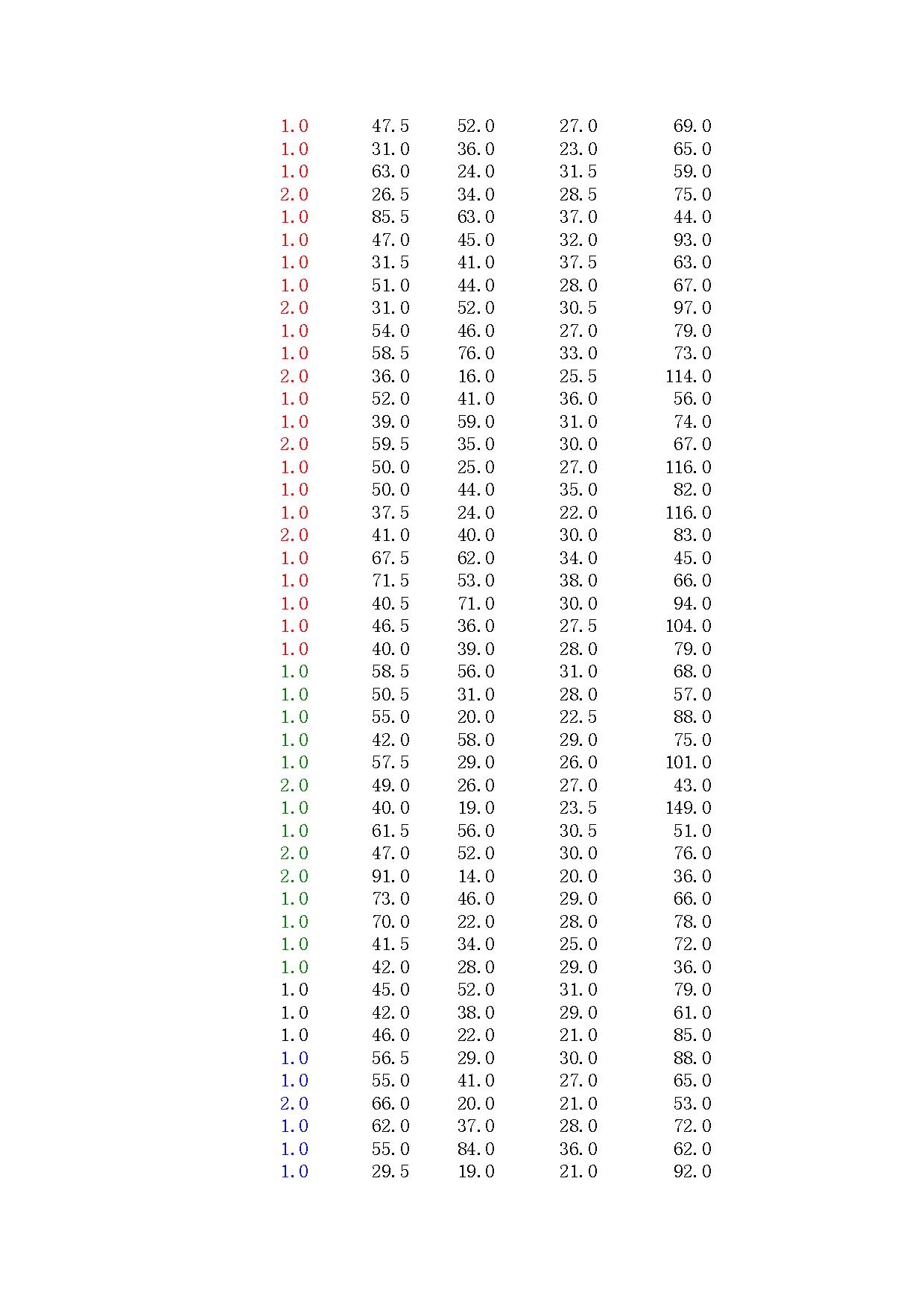

Supplement: Supplementary file 1 [file Data_Sheet_1.zip › Griffiths原始数据-4.jpg]

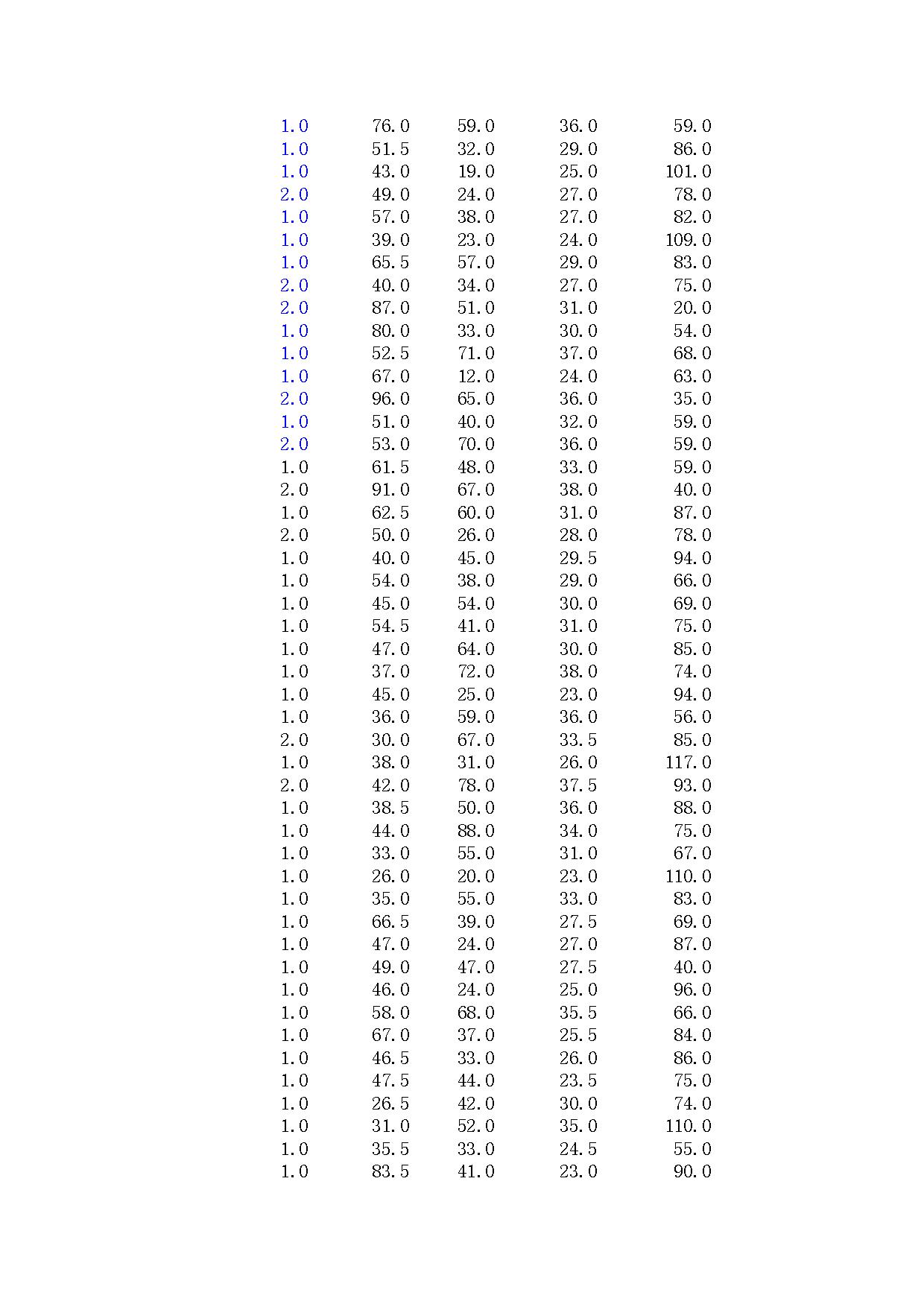

Supplement: Supplementary file 1 [file Data_Sheet_1.zip › Griffiths原始数据-5.jpg]

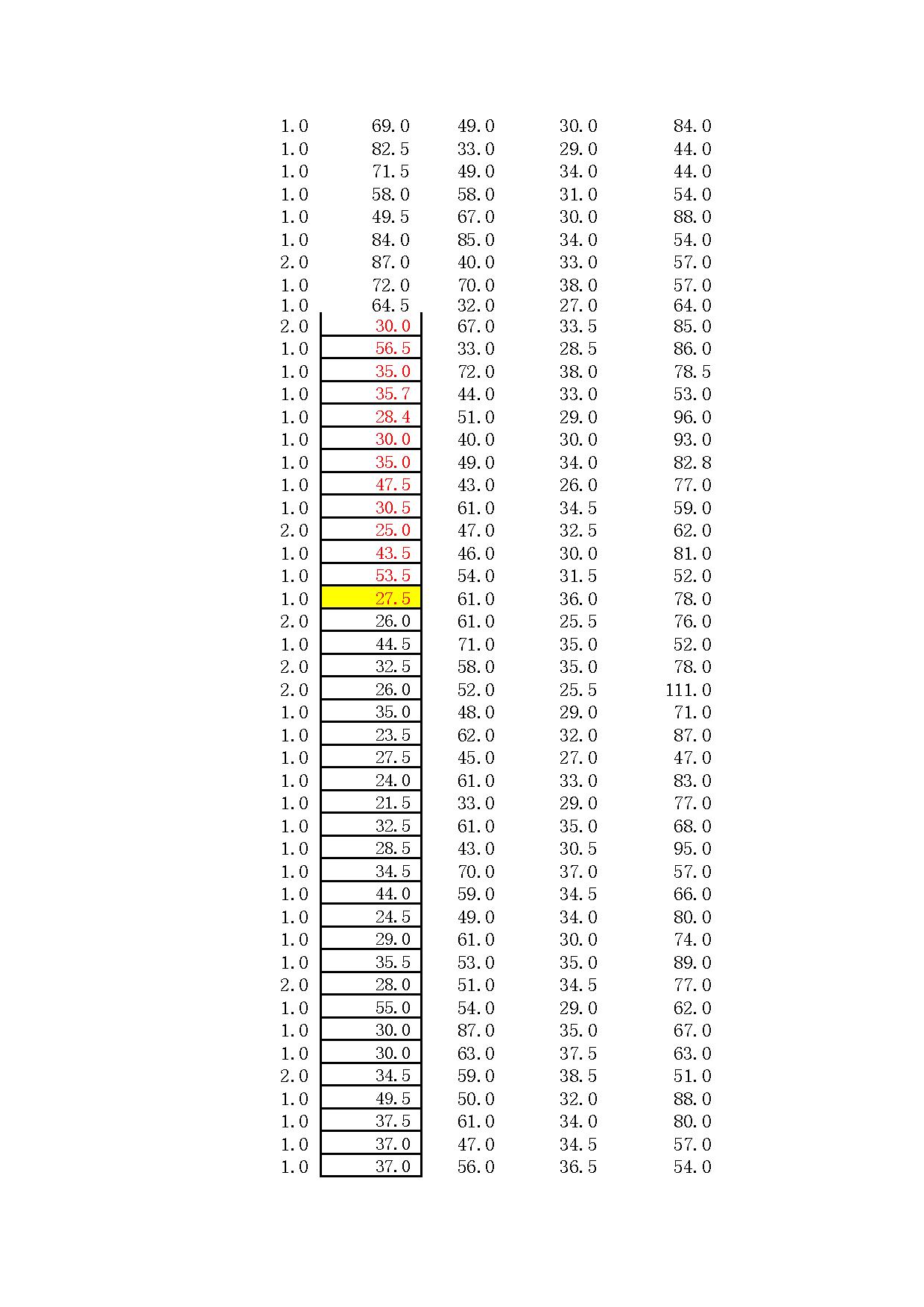

Supplement: Supplementary file 1 [file Data_Sheet_1.zip › Griffiths原始数据-6.jpg]

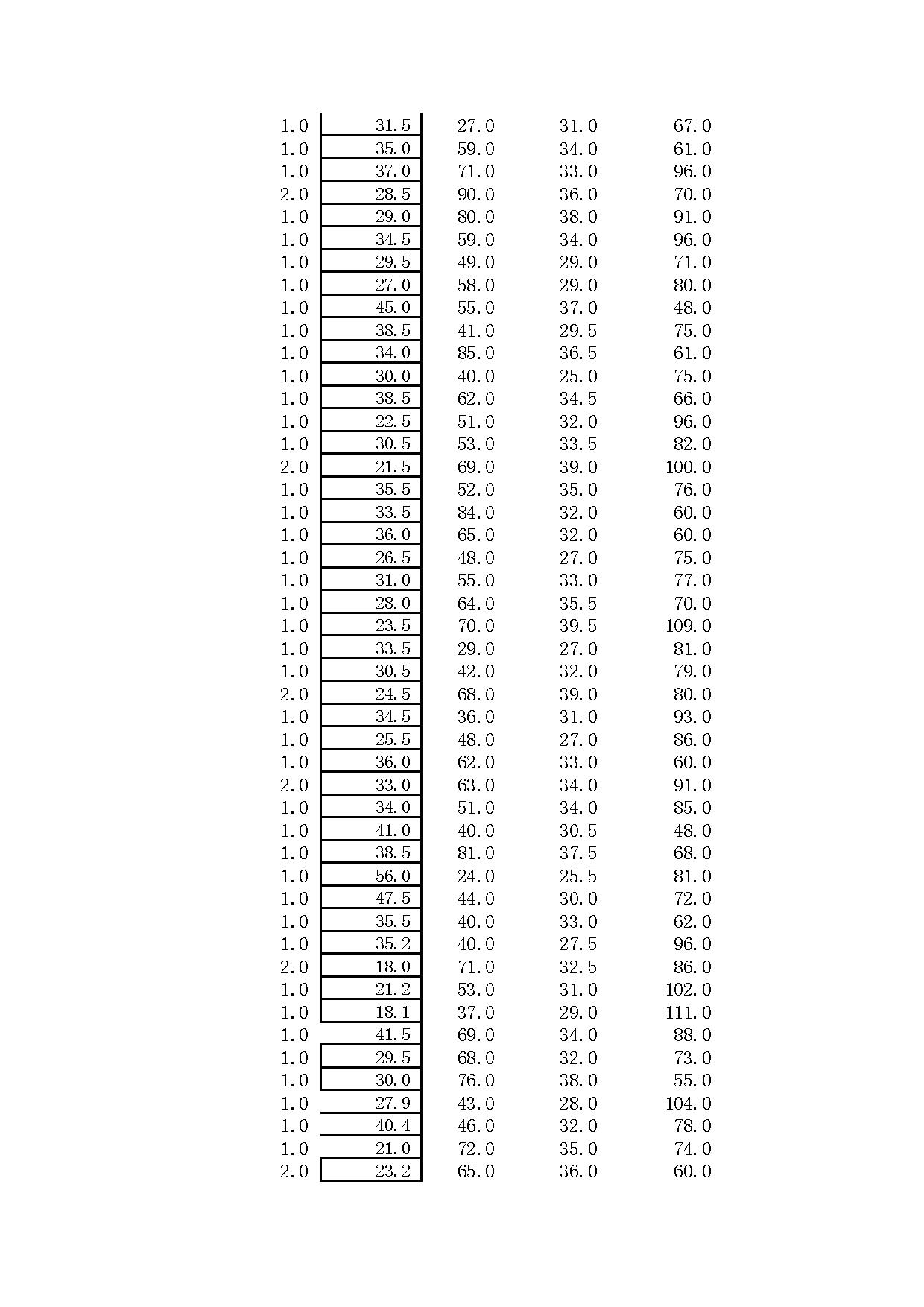

Supplement: Supplementary file 1 [file Data_Sheet_1.zip › Griffiths原始数据-7.jpg]

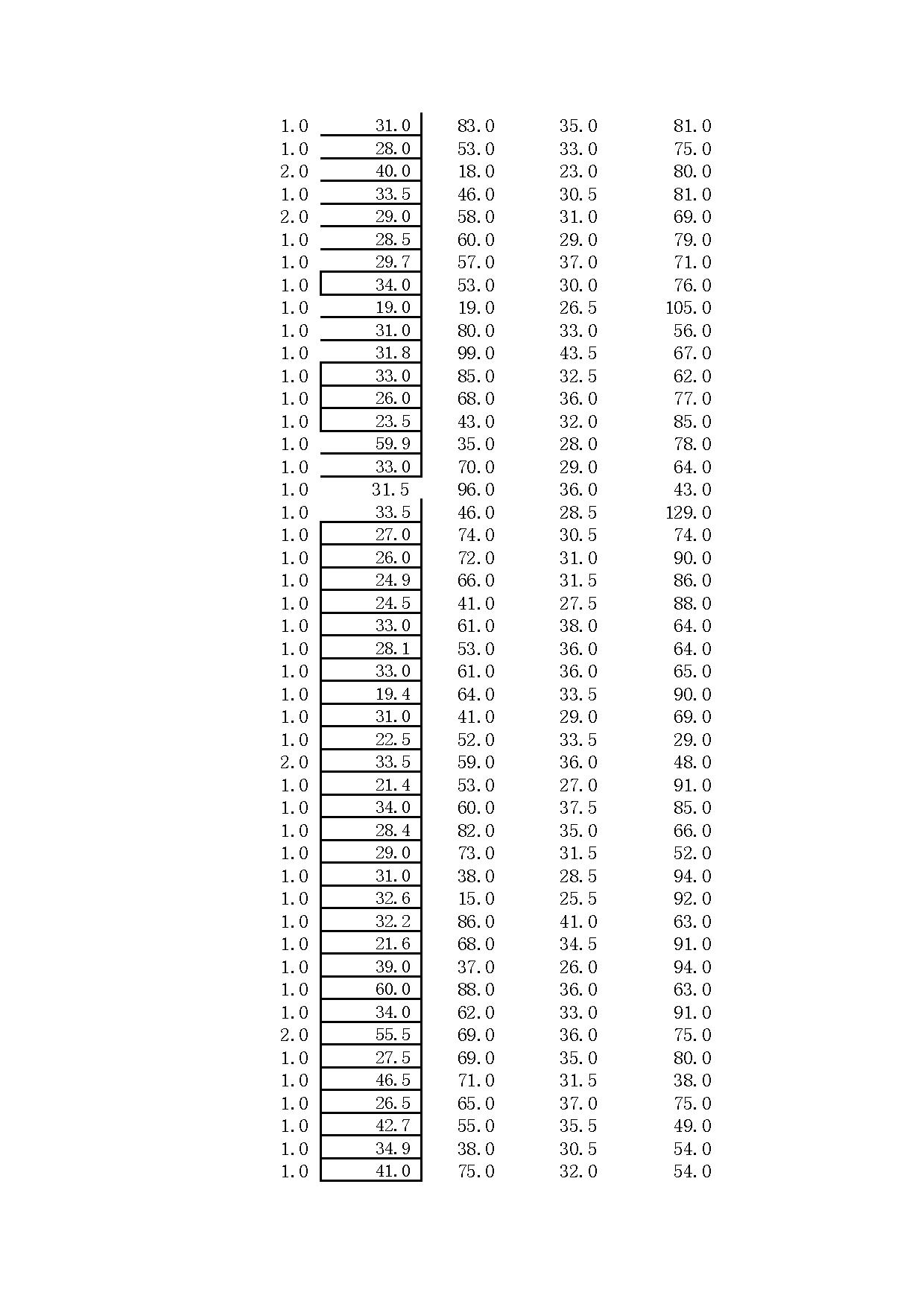

Supplement: Supplementary file 1 [file Data_Sheet_1.zip › Griffiths原始数据-8.jpg]

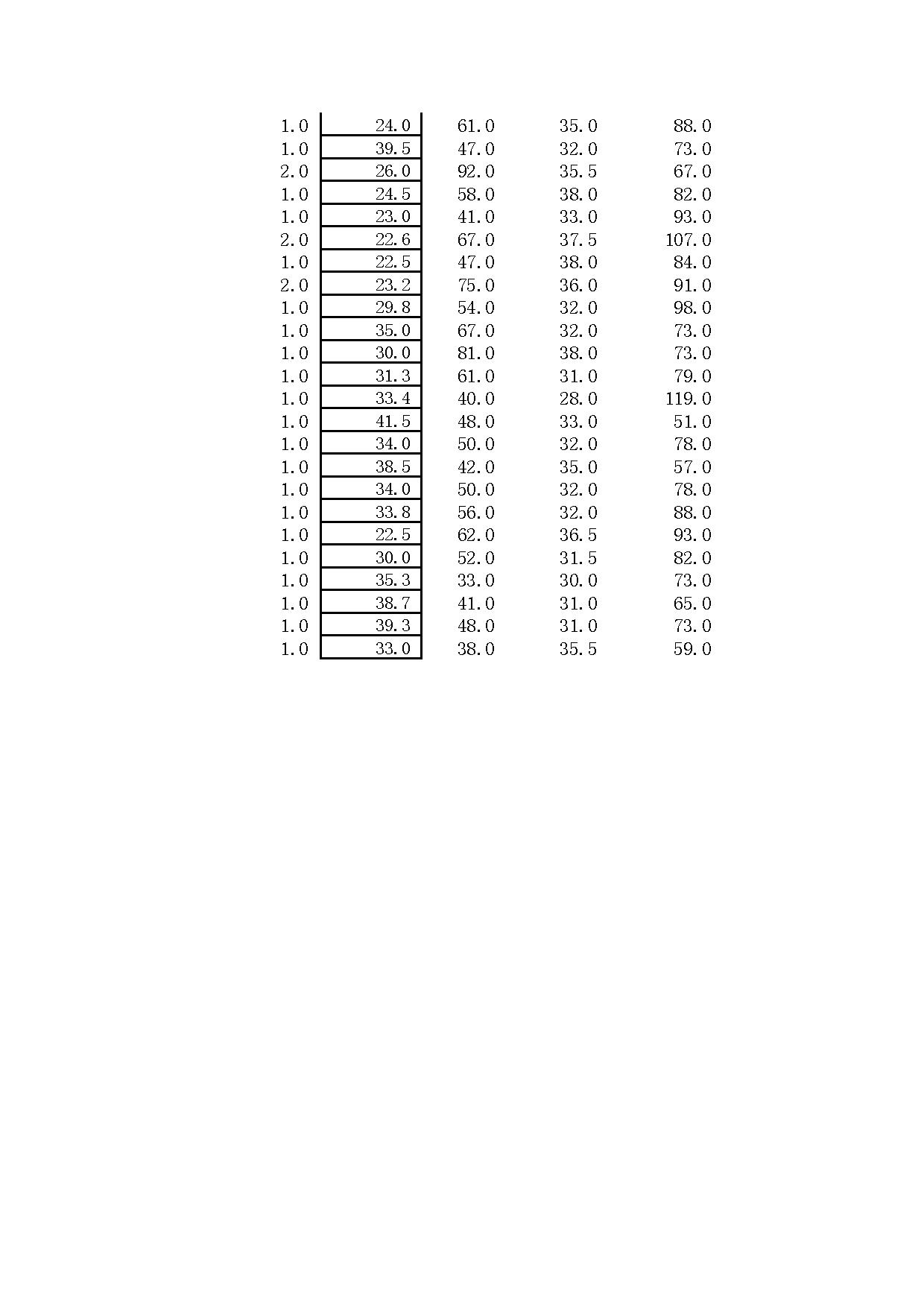

Supplement: Supplementary file 1 [file Data_Sheet_1.zip › Griffiths原始数据-9.jpg]
